# Dysfunction of STING Autophagy Degradation in Senescent Nucleus Pulposus Cells Accelerates Intervertebral Disc Degeneration

**DOI:** 10.7150/ijbs.88534

**Published:** 2024-04-08

**Authors:** Zhiqian Chen, Chen Chen, Xiao Yang, Yifan Zhou, Xiankun Cao, Chen Han, Tangjun Zhou, Jie Zhao, An Qin

**Affiliations:** Shanghai Key Laboratory of Orthopedic Implants, Department of Orthopedics, Ninth People's Hospital, Shanghai Jiaotong University School of Medicine, 639 Zhizaoju Road, Shanghai, 200011, P. R. China.

**Keywords:** STING Degradation, Autophagy, Intervertebral Disc Degeneration, Nucleus Pulposus Cells, Senescence.

## Abstract

The pathogenesis of Intervertebral Disc Degeneration (IDD) is complex and multifactorial, with cellular senescence of nucleus pulposus (NP) cells and inflammation playing major roles in the progression of IDD. The stimulator of interferon genes (STING) axis is a key mediator of inflammation during infection, cellular stress, and tissue damage. Here, we present a progressive increase in STING in senescent NP cells with the degradation disorder. The STING degradation function in normal NP cells can prevent IDD. However, the dysfunction of STING degradation through autophagy causes the accumulation and high expression of STING in senescent NP cells as well as inflammation continuous activation together significantly promotes IDD. In senescent NP cells and intervertebral discs (IVDs), we found that STING autophagy degradation was significantly lower than that of normal NP cells and IVDs when STING was activated by 2'3'-cGAMP. Also, the above phenomenon was found in STING^gt/gt^, cGAS^-/-^ mice with models of age-induced, lumbar instability-induced IDD as well as found in the rat caudal IVD puncture models. Taken together, we suggested that the promotion of STING autophagy degradation in senescent NP Cells demonstrated a potential therapeutic modality for the treatment of IDD.

## Introduction

Low back pain is a debilitating condition due to intervertebral disc degeneration (IDD), and usually precedes other spinal disorders including disc herniation, spondylosis, and lumbar spinal stenosis 1, 2. The intervertebral disc (IVD) is composed of an inner gel-like nucleus pulposus (NP) surrounded by annulus fibrosus (AF), and the cartilaginous end plates that connect to the adjacent vertebrae. Resident but scarce NP cells are responsible for the secretion of various extracellular matrix (ECM) proteins mainly including aggrecan and collagen II to maintain the intervertebral space height and provide physiological viscoelastic properties of the IVD to cushion axial mechanical pressure 3-5. Due to low cellularity, its avascular nature, and restricted nutrient transport, IVD is particularly susceptible to injury and the aging-associated accumulation of tissue damage, with limited repair potential leading to IDD 4, 6, 7. IDD is a highly catabolic process initiated within the central NP and characterized by extensive ECM degradation which progresses to the calcification and deterioration of NP architecture. Loss of disc height and space leads to the inability to maintain hydrostatic pressure and compression load 8. Premature cellular senescence, oxidative stress, and particularly chronic inflammation are omnipresent players and are intimately correlated to the progression of IDD 9. However, the underlying molecular players and mechanistic pathways that trigger these processes remain uncertain and therefore necessitate further research.

Genomic instability is a hallmark of age-related diseases with increased DNA damage considered a major instigator of cellular senescence and abnormal expression of inflammatory cytokines, a phenotype described as senescence-associated secretory phenotype (SASP) 10. Cytosolic DNA sensory system cyclic GMP-AMP synthase (cGAS)-stimulator of interferon genes (STING) pathway which is involved in cellular senescence, DNA damage, inflammatory response, and cell death 11. The cGAS is activated by binding to double-stranded DNA (dsDNA), including microbial dsDNA and nuclear and mitochondria-derived self-dsDNA 12, 13. This in turn triggers cGAS to generate the second messenger molecule 2'3'-cyclic GMP-AMP (2'3'-cGAMP), which binds and activates endoplasmic reticulum localized STING 14. During the translocation of activated STING to the Golgi, it recruits TANK-binding kinase 1 (TBK1) and inhibitory-κB kinase (IKK) to activate the IFN regulatory factor 3 (IRF3) and nuclear factor-κB (NF-κB) signaling pathway 15-17. This results in the elevation in the production of type I interferons and pro-inflammatory cytokines.

Some previous studies have confirmed the close relationship between STING and IDD. These researches confirm the critical roles of the cGAS-STING axis, cell death (apoptosis and pyroptosis), and inflammation in IDD 18-20. Lipopolysaccharide (LPS) induced vertebral inflammation of IDD models was also activated by the STING signaling 21. Recent research demonstrated that negative regulation of cGAS-STING through targeting STING for autophagy-lysosome degradation and ESCRT-dependent micro-autophagy 22, 23 attenuates the inflammation and decreases the immune response with cells attacked by the virus. However, the relationship between the expression changes of STING in normal and senescent NP cells and IDD has not been elucidated. Based on the above research and findings, we conducted the following study to explore the interaction of STING autophagy degradation, senescence of NP cells, and cGAS-STING axis in the progression of IDD.

## Materials and Methods

### Intervertebral disc specimens

Intervertebral disc (IVD) samples were obtained from patients undergoing routine lumbar spine surgery and IVD degeneration was evaluated using the Pfirrmann grading system based on T2-weighted images. Pfirrmann I IVD samples were obtained from three male patients, with an average age of 18.67 ± 1.76 years. These patients were presented with fresh traumatic lumbar fractures extending into the IVD and required discectomy and interbody fusion. Pfirrmann V IVDs were obtained from 3 male patients with an average age of 54.67 ± 4.06 years. These patients exhibited chronic intervertebral disc degeneration (IDD) with progressive neurologic discomfort and were unresponsive to conservative treatment thus requiring discectomy and interbody fusion intervention. Ethics approval was obtained from the Institutional Human Ethics Review Board of our hospital (Approval # SH9H-2020-T43-1). Written informed consent for the collection and use of tissue samples was obtained from all patients.

### Rat model of IDD

Fourteen 8-week-old male Sprague-Dawley rats were obtained from Shanghai Lab, Animal Research Center Co. Ltd (Shanghai, China) and acclimatized to the institute's animal facility before the surgical procedure. Rats were housed in a controlled environment of 22 ± 1ºC and relative humidity of 50 ± 1%, with a 12:12 hrs light/dark cycle. Rats were anesthetized by intraperitoneal injection of pentobarbital sodium (50 mg/kg of body weight) and their tail skin was sterilized with iodinated polyvinylpyrrolidone. The IVD was exposed via a dorsal skin incision. The tail caudal vertebral discs (Co6-10) were divided into Sham (Co6/7), Puncture only (Co7/8), and treatment (Co8/9 and Co9/10) groups. IVDs in puncture only and treatment groups were punctured with a 20-gauge sterile needle from the dorsal to the ventral side of the tail with the needle placed perpendicular to the skin to ensure that the needle was inserted (limited to 5 mm in depth) into the center of the nucleus pulposus (NP). Subsequently, Co8/9 IVDs were treated with 1μM H151 or 5 μM LDK378 or 100 μg/ml CMA, and Co9/10 IVDs with 5μM H151 or 10 μM LDK378 or 200 μg/ml CMA. Following treatment, the dorsal skin was sutured and disinfected again with iodinated polyvinylpyrrolidone. Rats were sacrificed at 12 weeks after surgery. The animal procedure was approved by the Animal Care and Ethics Committee of our hospital (Approval # SH9H-2021-TK326-1) and conducted following the regulations and guidelines of the institute.

### Murine model of intervertebral disc degeneration with aging and lumbar instability using STING^gt/gt^ and cGAS^-/-^ mice

The Golden-ticket mice (also known as Gt, Tmem173^I199N^, or STING^gt^) carry the I199N missense mutant allele of the STING gene. STING^gt/gt^ homozygotes do not produce type I IFNs in response to cyclic dinucleotides. STING^gt^ was heterozygous on a C57BL/6 background (Stock #: 017537) and was obtained from The Jackson Laboratory (Bar Harbour, ME, USA). cGAS^-/-^ heterozygous on a C57BL/6 background were obtained from the Nanjing Biomedical Research Institute of Nanjing University (Nanjing, China). Heterozygous mice were intercrossed to generate STING^gt/gt^ and cGAS^-/-^ homozygous mice. WT littermates were used as controls. For the aging model of IDD, IVDs were harvested from 3- and 22-month-old STING^gt/gt^ and WT mice. Simultaneously, discs of WT mice at 3, 12, and 24 months were obtained to examine the changes in the expression of STING with age. For the lumbar instability model, the spinous processes of L1-4 were resected from 12-week-old WT, STING^gt/gt,^ and cGAS^-/-^ mice, then the operative fields were closed with nylon sutures. All surgical procedures were performed under general anesthesia using 3% isoflurane approved by the Animal Care and Ethics Committee of our university and hospital and conducted under the regulations and guidelines of the institute. At 12 weeks post-surgery, mice were sacrificed by surgical dislocation, and the lumbar spine tissues were harvested for subsequent downstream analyses (L1-4). All mice were maintained under pathogen-free conditions.

### Murine model of intervertebral disc degeneration with premature aging using STING^gt/gt^ and ZMPSTE24^-/-^ mice

The ZMPSTE24^-/-^ mice were used for the establishment of the premature aging model which also induced intervertebral disc degeneration. ZMPSTE24^-/-^ heterozygous on a C57BL/6 background were obtained from Nanjing Biomedical Research Institute of Nanjing University (Nanjing, China). ZMPSTE24^-/-^ heterozygous mice and STING^gt^ heterozygous mice were intercrossed to generate STING^gt/gt^ homozygous, ZMPSTE24^-/-^ homozygous, STING^gt/gt^ -ZMPSTE24^-/-^ homozygous and wildtype mice. Due to the limited lifespan of ZMPSTE24^-/-^ homozygotes mice, all of the mice were sacrificed at 4 months old, and the lumbar spine tissues were harvested for further research (L1-4).

### Histology, immunofluorescence, and immunohistochemistry

Extracted IVD tissues from patients, rats, or mice were fixed in 4% paraformaldehyde (PFA) for 48 hrs, embedded into paraffin blocks, and processed for histological sectioning. Tissue sections 4 μm thick were stained with Safranin O-Fast Green (SOFG), Hematoxylin-Eosin (HE), and Sirius red. The degree of IDD was assessed by a histological scoring system, with scores ranging from 0 points (normal) to 15 points (severely degenerated). The scoring system is based on degenerative changes in the annulus fibrosus, the border between the annulus fibrosus and the nucleus pulposus, the parenchyma of the nucleus pulposus, and the matrix of the nucleus pulposus 24. For immunofluorescent staining, tissue sections were deparaffinized in graded xylene and standard alcohol gradient, and washed in PBS and water. Tissue sections were immersed in EDTA antigen retrieval buffer (pH 9.0) (Servicebio, Wuhan, China) and heated in a microwave oven for 10 mins at low heat. After natural cooling, sections were washed with PBS (pH 7.4), air-dried, and then an auto-fluorescence quencher was added for 5 mins. After a brief wash with running water for 10 mins, sections were blocked with BSA for 30 mins and then incubated in a wet box with anti-TOM20, anti-collagen II, anti-aggrecan, anti-MMP3, anti-dsDNA, anti-STING, anti-IL-1β, anti-TNF-α, anti LC3 or anti-IFN-β antibodies (1:100 dilution; Cell Signaling Technology and Abcam) overnight at 4°C. The next day, sections were washed with PBS and then incubated with Cy3-conjugated goat anti-mouse (1:300; GB21301) or Alexa Fluor® 488-conjugated goat anti-rabbit (1:400; GB25303) (Sevicebio) for 50 mins at RT in the dark. After extensive washing with PBS, sections were counterstained with DAPI for 3 mins at RT in the dark. Digital images were acquired with a fluorescence microscope (OLYMPUS CKX41).

### Radiographic and MRI analyses

Digital X-ray images of the caudal vertebrae (rats) or lumbar vertebrae (mice) were acquired in the anteroposterior axis using a Faxitron VersaVision equipped with 21 lp/mm detector that provides up to 5X geometric magnification (Faxitron Bioptics LLC, Tucson, AZ, USA). MRI scans were collected on a Siemens Magnetom Prisma E11 (Siemens Healthineers, Erlangen, Germany) with the following parameters for T2 mapping: TR 3000 ms, TE 80 ms, 1.1 mm thickness, 0.22 mm interval, FOV 160 mm × 65 mm, voxel size 0.25 mm × 0.25 mm × 1.1 mm in rat caudal vertebrae. Disc height indexes (DHI) were calculated by three spinal surgeons who were blinded to the treatment or control groups, where DHI = IVD height/adjacent IVD body height. IDD of the IVDs was also evaluated by three spinal surgeons using the Pfirrmann grading system based on T2 weight images of MRI scans.

### Micro-computed tomography analyses

Resected spinal tissues were fixed in a 4% PFA and micro-computed tomographic analysis was carried out on a SCANCO μCT100 scanner (SCANCO Medical AG, Brüttisellen, Switzerland) with the following scanning parameters: spatial resolution of 30 mm at the voltage of 70 kV and electrical current of 200 mA. Three-dimensional reconstructions were generated using the associated analysis software.

### Cell culture, STING overexpression, and knockout

The NP Cells were cultured in Dulbecco's modified Eagle's medium (DMEM) maintained with 10% FBS and 1% penicillin-streptomycin (Gibco, Thermo Fisher Scientific, Waltham, MA, USA) and incubated at 37°C, 21% O2, and 5% CO2 environment.

For STING overexpression of NP cells, the 293T cells were transduced with lentiviral vectors (pMD2.G and psPAX2) containing STING overexpression vectors for 48h. Then collect the virus-containing supernatant and immediately transfect NP cells with polybrene. Forty-eight hrs after transfecting, 5 μg/mL puromycin (Sigma-Aldrich) was used for select anti-puromycin NP cells and maintained thereafter with 1 μg/mL puromycin. The CRISPR-Cas9 technique was used to acquire STING knockout NP cells by amplifying the mono-clone of NP cells. Finally, western blotting was used to confirm overexpression and knockout efficiency.

### RNA isolation and qRT-PCR

The AxyPrep Total RNA Miniprep kit (Axygen, NY, USA) was used to isolate total RNA from tissue and cell samples under the manufacturer's instruction. RNA was then reverse transcribed into cDNA using PrimeScript™ 1^st^ Strand cDNA Synthesis Kit (TaKaRa Bio Inc., Shiga, Japan). Relative mRNA expression was determined using the GoTaq® 1-Step qRT-PCR system (Promega, Madison, WI, USA) and qPCR using SYBR® Premix Ex Taq Kit (TaKaRa Bio Inc.). The qRT-PCR reactions were performed on an ABI Prism 7500 Fast Real-Time PCR System (Applied Biosystems, CA, USA). The cycle threshold (Ct) values were obtained and normalized to the internal reference gene GAPDH. The comparative 2^-ΔΔCt^ method was used to calculate the relative mRNA expression of each target gene. Primer pairs were designed using Primer-BLAST and tabulated in the **Supplementary Table**.

### Protein extraction and western blot analyses

Total cellular proteins were extracted from treated NP cells using RIPA buffer supplemented with a protease inhibitor cocktail (Roche Holding AG, Basel, Switzerland). Lysates were cleared by centrifugation and the supernatant containing cellular proteins was retained. Protein concentrations were quantified using the Bicinchoninic Acid (BCA) Protein Quantification Kit (Thermo Fisher Scientific, Waltham, MA, USA), and an equal number of proteins (~25 μg) were resolved on 4-20% SDS-PAGE gel. Separated proteins were then electroblotted onto 0.22 μm PVDF membranes (Merck Millipore, Burlington, MA, USA). Membranes were blocked with 5% BSA in TBS-Tween (TBST) at room temperature (RT) for 1hr and then incubated with primary antibodies (1:1000 dilution in 5% BSA in TBST) at 4°C overnight. Primary antibodies used included anti-STING, anti-MMP3, anti-collagen II, anti-p65 and anti-phospho(p)-p65, anti-TBK1 and anti-p-TBK1, anti-IRF3 and anti-p-IRF3, anti-IFN-β, anti-IL-1β, anti-TNF-α, anti-P16, anti-P21, anti-P53, anti-Caspase 3, anti-PARP, anti-BAX, anti-BCL-2, anti-H2A, anti-γ-H2A, anti-GAPDH, and anti-β-actin (purchased from Cell Signaling Technology, Abcam and Affinity). The next day, membranes were washed 3 times with TBST and then incubated with anti-rabbit IRDye 800CW fluorescence secondary antibodies (LI-COR Biosciences, Lincoln, NE, USA) for 1hr at RT. The immunoreactive bands were detected using the Odyssey Infrared Imaging System (LI-COR Biosciences).

### Senescence assays

Senescent cells were identified using the Senescence β-Galactosidase Staining Kit (Cell Signaling Technology) following the manufacturer's instructions. Briefly, NP cells were treated with 2 μg/ml 2'3'-cGAMP, or 200 μg/ml CMA for 24 hrs and then cultured in normal DMEM for a further 3 days. NP cells overexpressing STING were cultured in DMEM for 3 days without stimulation. After 3 days of culture, cells were fixed with 1X Fixative Solution for 15 mins at RT and then incubated with β-Galactosidase Staining Solution at 37°C overnight in a dry incubator without CO_2_. Phase contrast images were captured under a light microscope (OLYMPUS CKX41).

### Comet Single Cell Gel Electrophoresis (SCGE) assay

Single Cell Electrophoresis Assay Kit (ENZO Life Sciences, Farmingdale, NY, USA) was used for comet analysis as per the manufacturer's protocol. Briefly, NP cells were treated without or with either 50 ng/ml IL-1β, 50ng/ml hydroxyurea, or 100 μM hydrogen peroxide (H_2_O_2_) for 24 hrs and then in normal DMEM for a further 3 days. Cells were then trypsinized, centrifuged, and resuspended in 1×PBS to a final density of 1×10^5^ cells per ml. 50 μl of cell suspension was mixed with 500 μl 1% low melting point (LMP) agarose, and then 75 μl of the cell-agarose mixture was spread onto glass slides pre-coated with 1% normal melting point agarose and allowed to settle for 10 mins at 4°C in the dark. Slides were then incubated in pre-chilled Lysis Solution on ice for 1 hr and then placed on a horizontal gel electrophoresis unit containing electrophoretic Alkaline Solution for 60 mins at RT in the dark to allow the DNA to unwind. Electrophoresis was carried at 25 V and 300 mA (0.73 V/cm) for 10 mins. After electrophoresis, slides were rinsed in distilled water, placed in a Neutralization Solution (pH 7.5), dehydrated with 70% ethanol for 5 minutes, and air-dried. Before the examination, slides were stained with 100 μl CYGREEN® Dye for 30 mins at RT in the dark. Allow the slides to dry completely at 37°C and view them by fluorescence microscopy (OLYMPUS CKX41) with excitation and emission wavelengths of 489 and 515 nm respectively.

### Isolation of mitochondrial DNA (mtDNA) and nuclear DNA (nDNA)

Mitochondrial (mt) DNA and nuclear (n) DNA were isolated as previously described. Briefly, NP cells treated without or with either 50 ng/ml IL-1β, 50ng/ml hydroxyurea, or 100 μM hydrogen peroxide (H_2_O_2_) for a 24 hrs and then in normal DMEM for a further 3 days were lysed in Digitonin buffer to isolate dsDNA in the cytoplasm from the supernatant. Then NP40 buffer is used for the extraction of DNA from the nucleus in the pellet. Real-time qPCR was used to quantify mitochondrial DNA and nuclear DNA in the cytoplasm. To more accurately describe the changes of mtDNA and nDNA in the cytoplasm, The following mitochondrial genes were examined: *mtND1*, *mtCYTB*, and *mtCOX1*. The following nuclear genes were examined: *ACTB*, *GUSB*, and *B2M.*

### Assessment of apoptosis by flow cytometry

To clarify the impacts of activation of STING, the NP cells were stained with APC-Annexin V and propidium iodide (PI) by using an apoptosis staining kit (Thermo Fisher Scientific) and PI/RNase Staining Buffer (BD Pharmingen) based on the manufacturer's protocol. At least 10,000 cell suspensions were detected by flow cytometry on a FACS Calibur Flow Cytometer (BD Biosciences). The apoptotic rate of NP cells was quantified based on the right upper (Q2; positive staining for APC-Annexin V and PI) and right lower (Q3; positive staining for APC-Annexin V and negative for PI) quadrant according to the flow cytometric scatterplot.

### High-density culture

Resuspending about 150,000 NP primary cells in 10 μl culture medium, and seeded in the form of micro-masses in the bottom of a 12-well plate. After 1 h at 37°C, the NP primary cells were attached to the bottom of culture plates; then added 1 ml of MEM/F12 medium containing 10 ng/ml of ITS (Insulin Transferrin Selenium) and 2% FBS. The medium was refreshed every two days. These micro-masses were stained with Alcian blue after 9 days.

### NP Primary Cells Isolation

The NP tissues were obtained from SD rat (4-weeks-old) caudal discs at Co1 to Co6. Those tissues were digested with 0.25% type II collagenase for 2 hrs in a CO2 incubator. Then, the treated NP cells were resuspended and cultured with DMEM including 10% FBS and 1% penicillin-streptomycin (Gibco, Thermo Fisher Scientific, Waltham, MA, United States) after centrifugation. All the cells were incubated at 37°C, 21% O2, and 5% CO2 environment. All the experimental procedures were approved by the Animal Care and Ethics Committee of our university and conducted following the regulations and guidelines of the institute.

### Single-cell sequencing database

The sample details of single-cell sequencing data were available in the GEO database, GSE205535 (Zhencong Li et al., 2022). The expression of DNA markers in degenerative and normal NP cells was shown by VlnPlot and cluster analysis. A p-value of less than 0.05 was considered statistically significant between the degenerative and normal NP groups.

### TUNEL staining

TUNEL staining was performed using the "*In situ* cell death detection" Kit (Roche Diagnostic). Briefly, the NP cell samples or IVDs samples with air-dried were fixed by 4% PFA then closed and used permeable fluid to promote permeability. Drop TdT enzyme reaction solution to make NP cells acquire fluorescence label. Finally, using a fluorescence microscope (OLYMPUS CKX41) detect TUNEL staining.

### Statistical analysis

All data are presented as the mean ± standard deviation (S.D.). Significance differences between study groups were obtained by Student's *t*-test or one-way analysis of variance (ANOVA) using GraphPad Prism 7 Software (GraphPad Software, San Diego, CA, USA). A p-value < 0.05 were considered statistically significant.

## Results

### Elevated cytoplasmic dsDNA release during IDD

**Figure 1:**
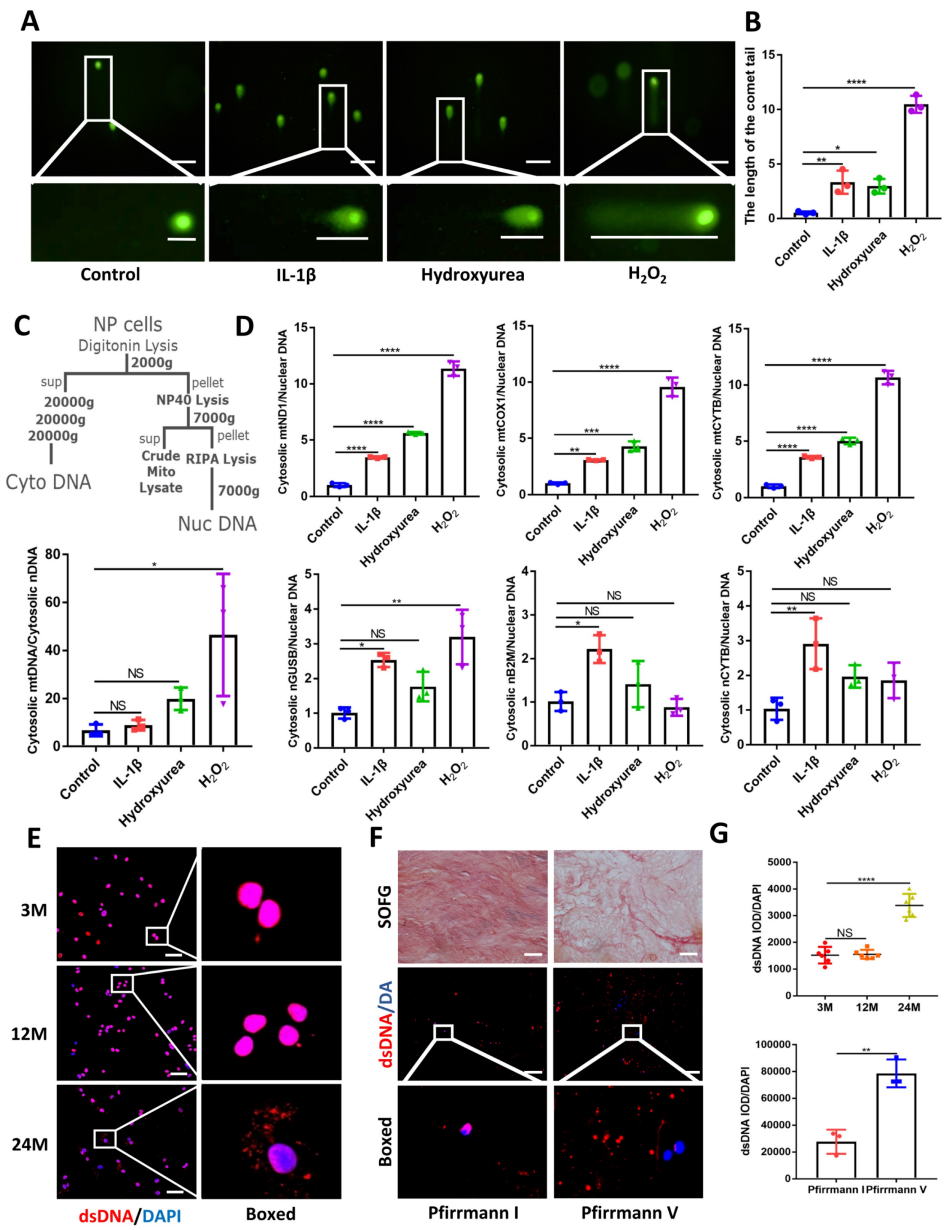
** Elevated dsDNA release in degenerative NP cells. (A)** Comet tailing experiment demonstrated the degree of DNA damage of NP cells after being stimulated with IL-1β, hydrogen peroxide, and hydroxyurea. (Scale bars= 100 µm.) **(B)** Statistics of the distance of the comet tail. **(C)** Schematic of subcellular fractionation including mtDNA and nDNA in the cytoplasm and the Quantitative real-time PCR (qPCR) demonstrate the ratio of mtDNA and nDNA in the cytoplasm. **(D)** The qPCR analysis of mtDNA including mtND1, mtCOX1, and mtCYTB as well as nDNA including nGUSB, nB2M, and nACTB in the cytoplasm. **(E)** Immunofluorescence examined the changes of dsDNA during IDD in 3-,12-, and 24-month WT mice. (Scale bars=50 µm.)** (F)** Safranin O-Fast Green (SOFG) staining and immunofluorescence detected the release of dsDNA in human NP tissues of Pfirrmann I and Pfirrmann V. (Scale bars=50 µm.) **(G)** The fluorescence statistics of the cytoplasm-released dsDNA in (E) and (F). (The cells used were the rat NP cell line. The spinal level used was L4/5 level. Data were expressed as mean±SD. *p<0.05; **p<0.01; ***p<0.001; ****p<0.0001).

Using comet tailing experiments, significantly induced DNA damage was found in treated NP cells with IL-1β, hydroxyurea, and hydrogen peroxide (H_2_O_2_)** (Figure 1A and 1B)**. Additionally, immunofluorescence images showed elevated release of dsDNA into the cytoplasm compared with NP control cells **(Figure S1A)**. In **Figure 1C, 1D**, we demonstrated that an elevation in the ratio of mtDNA to nDNA in the cytoplasm is consistent with the increase in mitochondrial genes such as mtND1, mtCOX1, and mtCYTB in the cytoplasm. Concerning the presence of nDNA in the cytoplasm, treatment with IL-1β showed consistent elevation in cytoplasmic nGUSB, nB2M, and nACTB expression compared to the control. The nDNA in the other treated groups of cytoplasm also showed an upward trend. All the above data showed that the release of dsDNA (both mtDNA and nDNA) in cytoplasm elevated in degenerative NP cells caused by inflammation, senescence, and oxidative stress.

We next examined the release of dsDNA during aging and IDD. The dsDNA was examined by histology and immunofluorescence at 3, 12, and 24 months in WT mice. The progressive degeneration of the NP was observed with aging and severe IDD was observed in 24-month-old mice** (Figure 1E, 1G and Figure S2A, S2B)**. To further demonstrate the degree of elevated cytoplasmic dsDNA with IDD, we examined human degenerative IVDs (Graded as Pfirrmann I and Pfirrmann V). Safranin O-Fast Green (SOFG) showed less ECM in Pfirrmann V graded IVDs which also correlated with elevated leakage of cytoplasmic dsDNA in the NP when compared with Pfirrmann I graded IVDs **(Figure 1F and 1G)**. These data suggest that elevated cytoplasmic dsDNA is positively correlated with age-induced IDD.

### The deficiency of DNA sensor cGAS rescued mice from IDD

**Figure 2:**
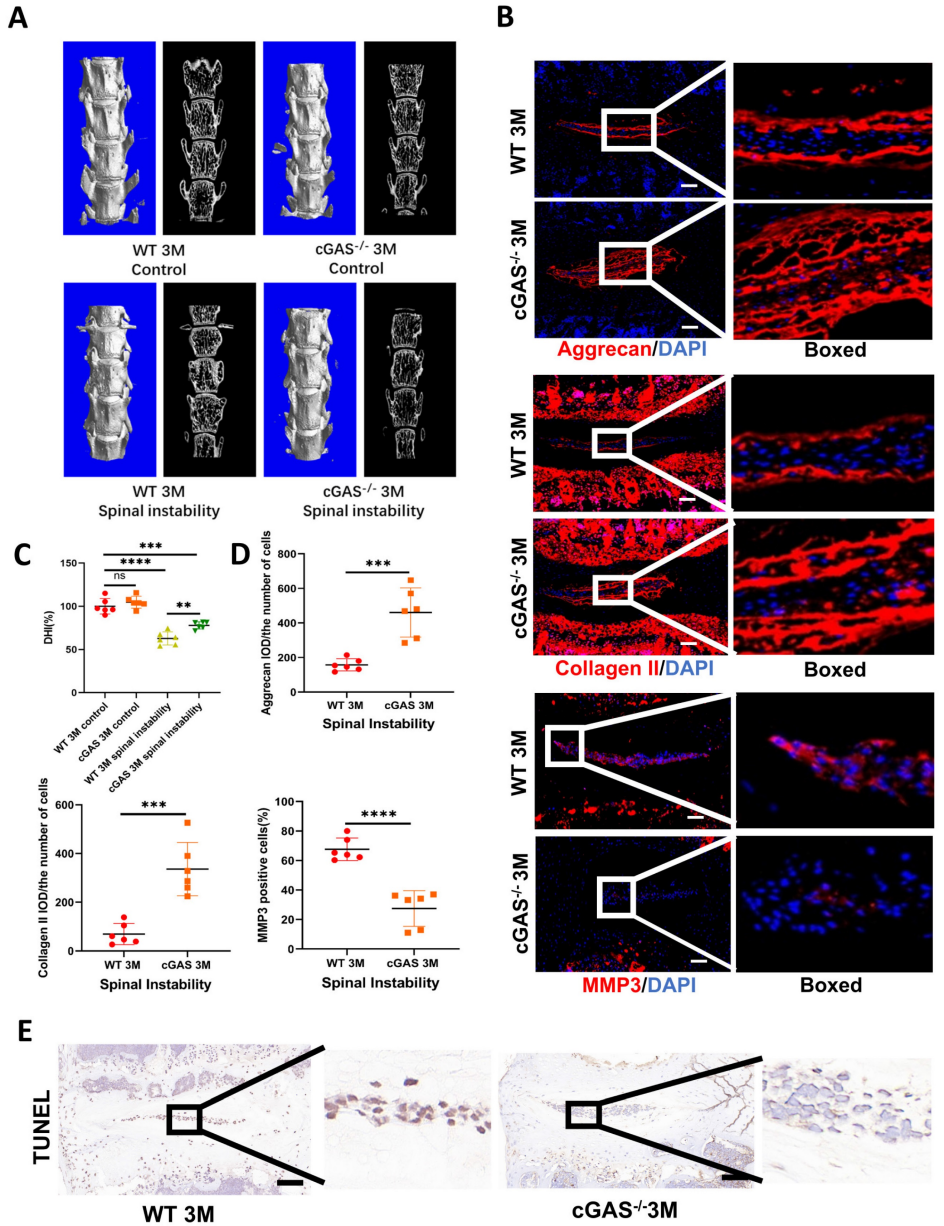
** cGAS knock-out protects mice from instability-induced IDD. (A)** Micro-CT of 3D reconstruction showed the disc height of lumbar vertebra instability models.** (B)** Immunofluorescence examined the changes of aggrecan, collagen II, and MMP3 in IVDs with spinal instability in WT and cGAS^-/-^ mice. **(C)** Quantitative analysis of the DHI% in two groups of vertebral instability models.** (D)** Statistics of (B) in two groups of vertebral instability mice. (E) The TUNEL tests showed reduced apoptosis in the intervertebral discs of cGAS^-/-^ mice in the instability models (Scale bar=50μm. The spinal level used was L4/5 level. Data are expressed as mean±SD. ***p<0.001; ****p<0.0001).

The cGAS is the major sensor of cytosolic dsDNA. We first examined the function of cGAS on IDD induced by instability. Severe degeneration of the IVDs was observed in WT mice compared with cGAS^-/-^ mice with Hematoxylin-eosin (HE) and SOFG **(Figure S3A and S3B)**. X-ray, micro-CT 3D reconstruction, and coronal plane showed the disc height was protected to a certain extent in the cGAS^-/-^ mice **(Figure 2A, 2C, S3C, and S3D)**. Simultaneously, more ECM (aggrecan and collagen II) and fewer catabolic maker (MMP3) was observed in the instability model of cGAS^-/-^ mice **(Figure 2B and 2D)**. STING expression was still elevated in the instability model of cGAS-/- mice, but failure to activate STING resulted in the protection of apoptosis in IVDs** (Figure S3E and 2E).** Thus, the blockage of DNA sensor cGAS has the function of postponing the IDD process.

### Up-regulated STING expression in degenerative NP cells

**Figure 3:**
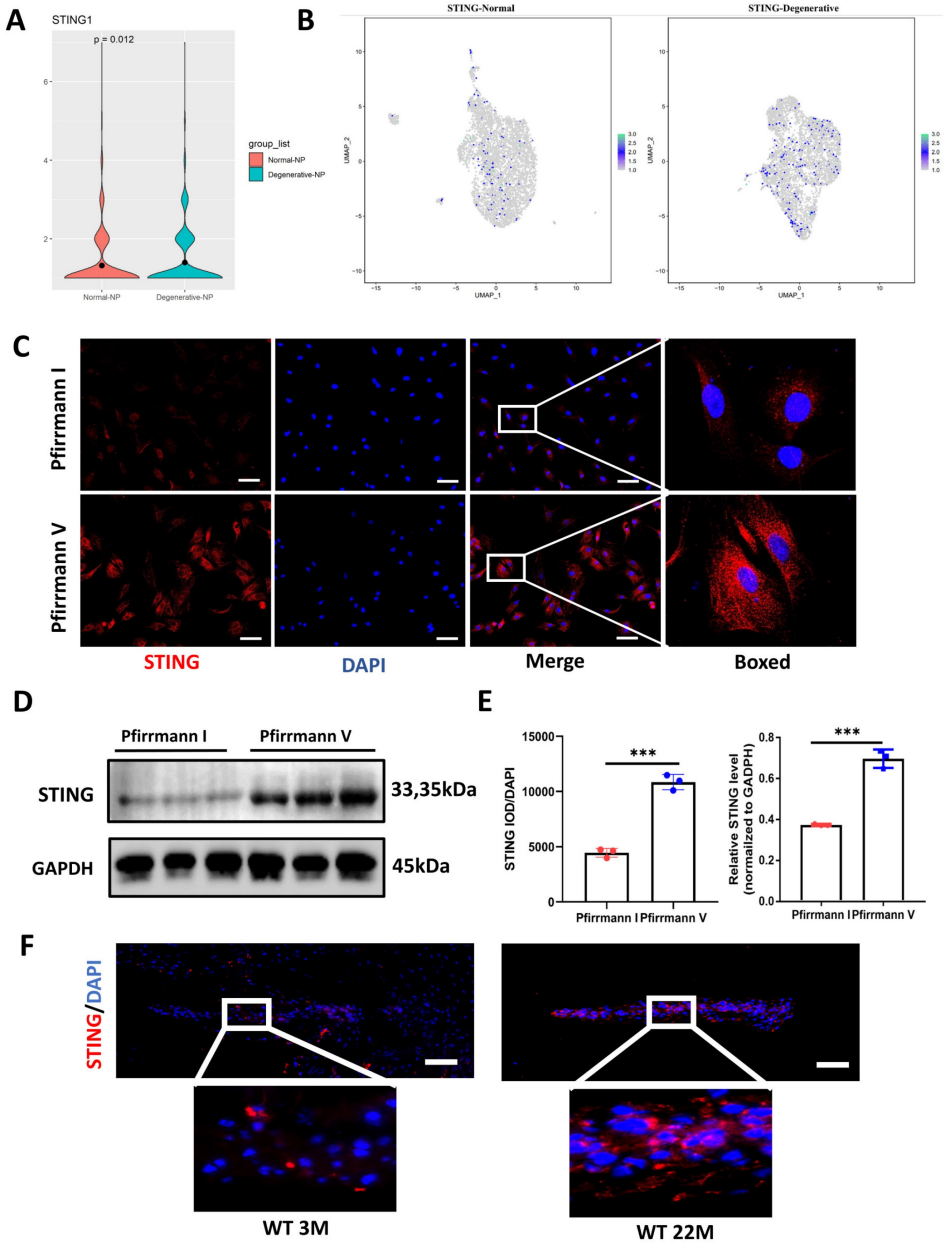
**Elevated expression of STING in the degenerative intervertebral disc. (A)** Single-cell sequencing showed the up-regulated STING in normal and degenerative human NP cells. **(B)** Cluster analysis showed that STING was significantly increased in normal and degenerated human nucleus pulposus. **(C, D)** Immunofluorescence and western blot examined the changes of STING during IDD in Pfirrmann I and Pfirrmann V nucleus pulposus. **(E)** Quantitative analysis of (C) and (D) (Scale bar=20μm.). (F) Immunofluorescence demonstrated that STING expression increased significantly in the intervertebral discs of aged mice. (Scale bar=20μm.) (The NP cells and tissues used are of human origin. Data are expressed as mean±SD. ***P<0.001 compared with controls).

**Figure S4** mapped out the gene network most closely related to STING by using Cytoscape. cGAS, IRF3, and TBK1 did not change significantly between the degenerative and normal NP cells **(Figure S4B)**. Moreover, the up-regulated STING expression in degenerative NP cells by cluster analysis and violin plot was shown in **Figure 3A and 3B**. At the protein expression level, a marked elevation of STING expression was found in Pfirrmann V IVDs compared to Pfirrmann I IVDs **(Figure 3C, 3D, and 3E)**. A significant increase in STING was observed in the intervertebral disc of 22-month-old mice** (Figure 3F)**. The above results demonstrated that STING is increased expression in degenerative NP cells.

### Senescent NP cells have the characteristic of STING autophagy degradation obstacle causing the accumulation of STING and forming a vicious circle

**Figure 4:**
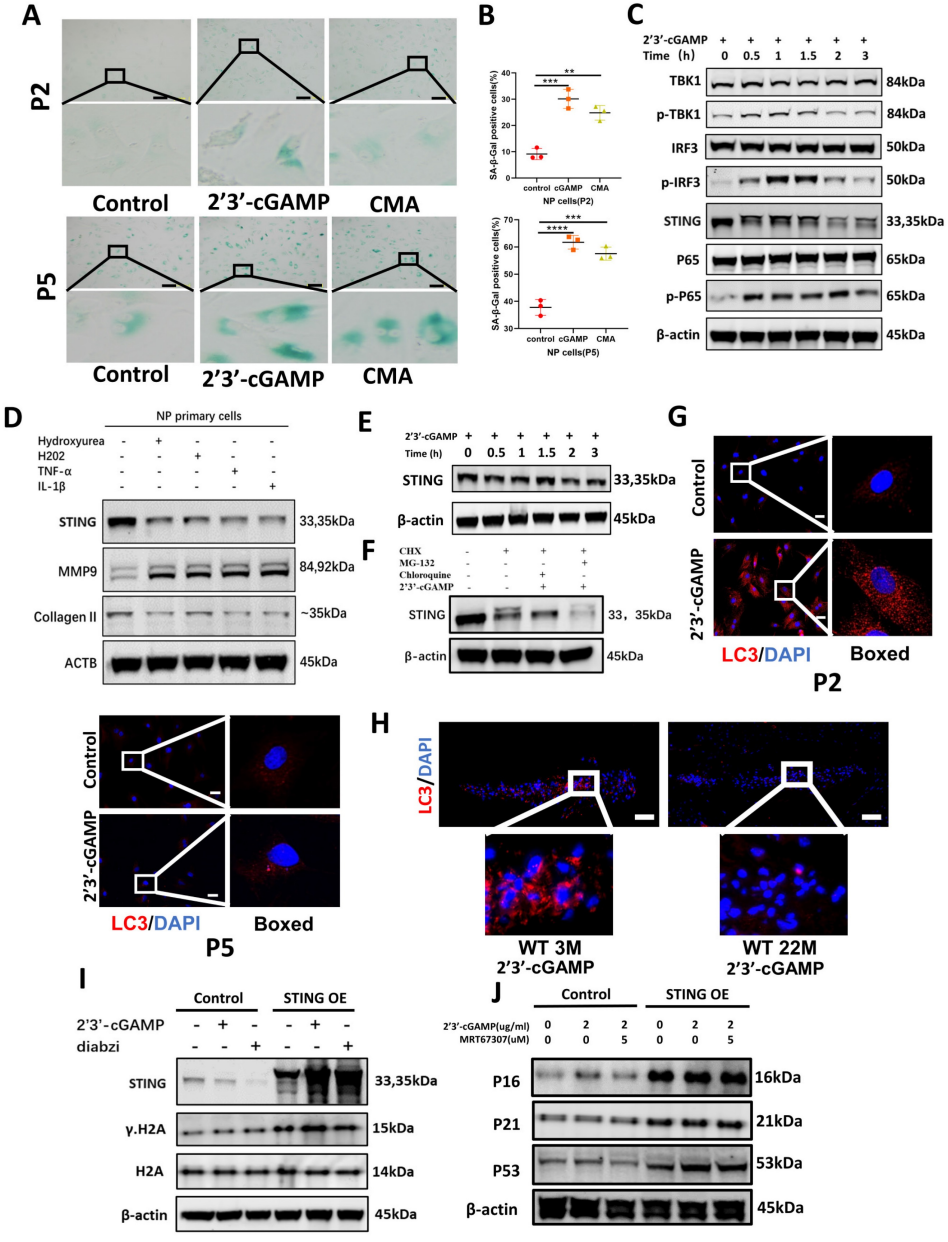
** The decrease of autophagy in senescent NP cells causes the accumulation of STING creating a vicious circle. (A-B)** The Sa-β-Gal assay showed the senescence of primary NP cells after being treated with 2'3'-cGAMP, and CMA in P2 and P5 generation. **(B)** Quantitative analysis of the proportion of primary NP cells in the β-Gal positive. **(C)** Western blot showed STING, phosphorylation of P65, TBK1and IRF3 changed with time after being treated with 2'3'-cGAMP for 0min, 30min, 1h, 1.5hrs,2hrs, and 3hrs in P2 NP cells.** (D)** Western blot examined the expression of STING, MMP9, and Collagen II in NP primary cells after being treated with dsDNA extracted from IL-1β, hydrogen peroxide, and hydroxyurea-treated supernate.** (E)** Western blot showed STING changes after being stimulated with 2'3'-cGAMP for 0min, 30min, 1hrs, 1.5hrs,2hrs, and 3hrs in P5 NP cells. **(F)** Changes of the expression of STING after being treated with CHX, CHX+chloroquine+2'3'-cGAMP, or CHX+MG-132+2'3'-cGAMP P2 NP cells.** (G)** Immunofluorescence showed the activation of LC3 with the 2'3'-cGAMP in P2 and P5 NP cells. **(H)** Immunofluorescence examined the expression of LC3 in the intervertebral discs of 3M- and 22M- mice.** (I)** Immunoblot showed the changes of STING, γ-H2A, and H2A after treating with 2'3'-cGAMP or diabzi for 4 hrs in normal and STING over-expression NP cell lines.** (J)**Immunoblot showed changes of P16, P21, and P53 after treatment with 2'3'-cGAMP and co-stimulated 2'3'-cGAMP with MRT67307 for 48 hrs in the NP cell line. (The concentration of 2'3'-cGAMP (2ug/ml), CMA (100ug/ml), MRT67307(2.5uM) and diabzi (500nM), CHX (0.5 μM), MG132 (5μM), chloroquine (10μM) was treated NP cells. Scale bar=50μm. Data are expressed as mean±SD, **p<0.01, ***P<0.001 and ****p<0.0001 compared with controls).

Based on the close connection between senescence and IDD. The Measurement of lysosomal β-galactosidase activity further found that STING induction leads to premature senescence of NP primary cells in P2 and P5 generation **(Figure 4A and 4B)**. The activation of inflammatory pathways including TBK1-IRF3 and NF-κB signaling pathways **(Figure 4C)** is more intense under the condition of STING overexpression **(Figure S5B)**. The STING degradation was found in P2 NP cells after STING activation **(Figure 4C)** and this phenomenon disappeared in senescent NP cells at P5 generation **(Figure 4E)**. Also, the expression of STING was degraded after being treated with the cytoplasmic dsDNA extracted from the supernate of P2 NP cells treated by IL-1β, hydrogen peroxide, and hydroxyurea **(Figure 4D)**. Chloroquine ameliorates the STING degradation treated by 2'3'-cGAMP **(Figure 4F).** LC3 dots represent that autophagy activation was more obvious in P2 NP cells compared with senescent NP cells **(Figure 4G).** Fewer LC3 dots in the IVDs of 22-month-old mice after being stimulated with 2'3'-cGAMP compared with 3-month-old mice demonstrated that the STING autophagy degradation became less (**Figure 4H**)**.** In STING overexpression NP cells treated with 2'3'-cGAMP or diabzi, γ.H2A was up-regulated which indicated the senescence has been significantly aggravated **(Figure 4I)**. The marked upregulation of p53, p21, and p16 was found after stimulation in STING overexpression NP cells **(Figure 4J)**. Taken together, the senescent NP cells have the obstacle of STING autophagy degradation that causes the STING accumulation, the activation of inflammation, and senescence pathways.

### The accumulation of STING promotes apoptosis in NP cells

**Figure 5:**
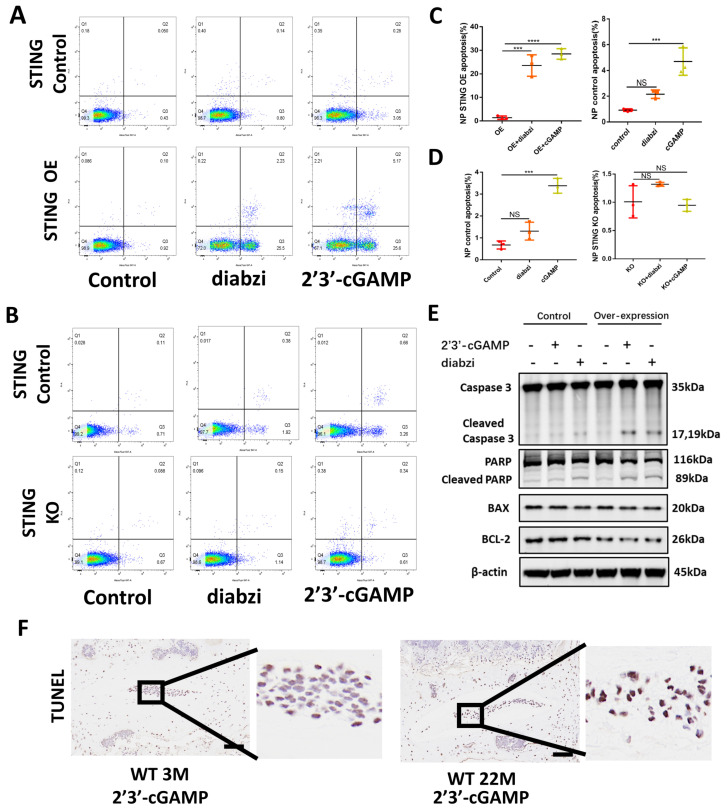
** Accumulation STING in senescence of NP cells promoted apoptosis. (A, B)** Fluorescence-activated cell sorting examined the number of apoptotic cells after activation with 2'3'-cGAMP and diabzi in control, STING high-expression, and STING knock-out NP cells. **(C, D)** Statistics of apoptotic cells in fluorescence-activated cells.** (E)** Immunoblot detected the changes of apoptosis factors concluded caspase 3, cleaved-caspase 3, PARP, cleaved-PARP, BAX, and Bcl-2 after treatment with 2'3'-cGAMP or diabzi for 24 hrs in normal or STING over-expression NP cells. **(F)** TUNEL tests examined the apoptosis in NP cells after stimulated with 2'3'-cGAMP in 3M- and 22M- mice (The cells used were the rat NP cell line. Scale bar=50μm. Data are expressed as mean±SD, ***P<0.001, and ****p<0.0001 compared with controls).

The apoptosis of NP cells in control, STING knockout, and STING overexpression groups stimulated by 2'3'-cGAMP or diabzi was detected by TUNEL assay **(Figure S5A and S5D)** and flow cytometry analysis **(Figure 5A and 5C)**. The results showed a significant increase in apoptosis was observed in STING over-expression NP cells. On the contrary, the apoptosis was partially abolished in normal or STING knockout NP cells **(Figure 5B and 5D)**. The activated STING promoted the apoptosis of NP cells with up-regulated cleaved-caspase3, cleaved-PARP, and down-regulated BCL2 in STING OE NP cells **(Figure 5E)**. More apoptosis in NP cells was found in senescent NP cells of IVDs after being stimulated with 2'3'-cGAMP in 22-month mice **(Figure 5F).** These results suggest that the activation of accumulation STING significantly enhanced the apoptosis of nucleus pulposus cells.

### Inhibition of the function of STING alleviated IDD

**Figure 6:**
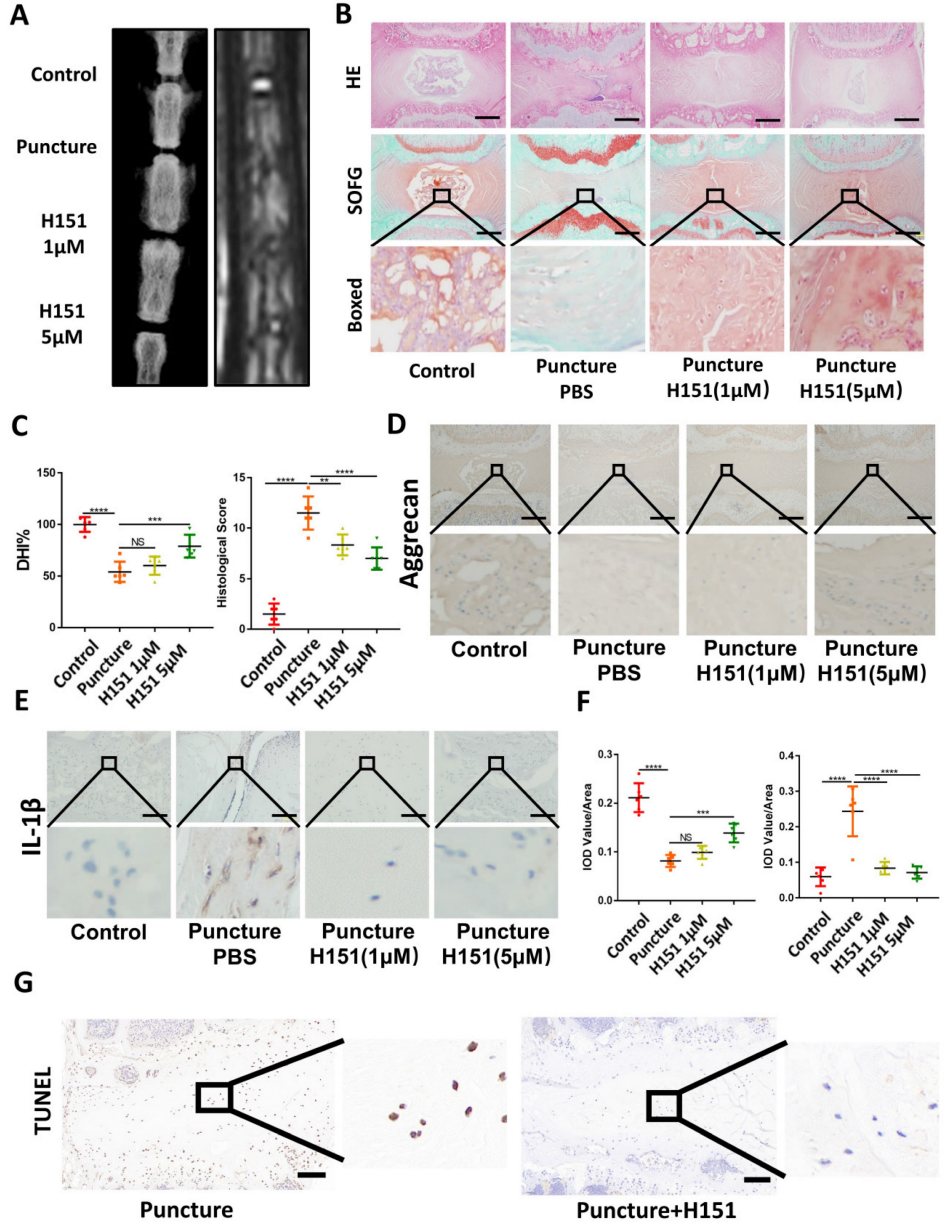
** Inhibition of STING alleviated IDD in the rat model. (A)** X-ray and MRI showed the changes of the IVDs in the control, puncture, H151 1μM, and H151 5μM group. **(B)** HE and SOFG staining of the IVDs in 4 groups of caudal vertebrae puncture rat models. **(C)** The quantitative analysis of DHI% in (A) and histological score in (B). **(D-E)** Immunohistochemistry detected the changes of aggrecan and IL-1β in the control, puncture, H151 1μM, and H151 5μM IVDs.** (F)** The quantitative analysis of aggrecan and IL-1β IOD Value/Area in (D) and (E) respectively. **(G)** The apoptosis of NP cells was detected by TUNEL tests in the control and H151 5μM IVDs. (The spinal level used was Co6-10 level. Scale bar=50μm. Data are expressed as mean±SD, **p<0.01, ***P<0.001, and ****p<0.0001 compared with controls).

X-ray and MRI analysis showed that H151 administration minimized osteophyte formation, maintained IVD height, and prevented the collapse of the IVD space **(Figure 6A and 6C)**. HE and SOFG of the IVDs showed that H151 treatment helped preserve the morphological structure and matrix composition of the annulus fibrosus (AF) and nucleus pulposus (NP) **(Figure 6B and 6C)**. Immunohistochemistry revealed that inhibition of STING reduced the degradation of aggrecan and hindered the secretion of inflammatory factors containing IL-1β **(Figure 6D, 6E, and 6F)**. The activation of STING-caused apoptosis of NP cells was inhibited after the use of H151 **(Figure 6G and S4C)**.

**Figure 7:**
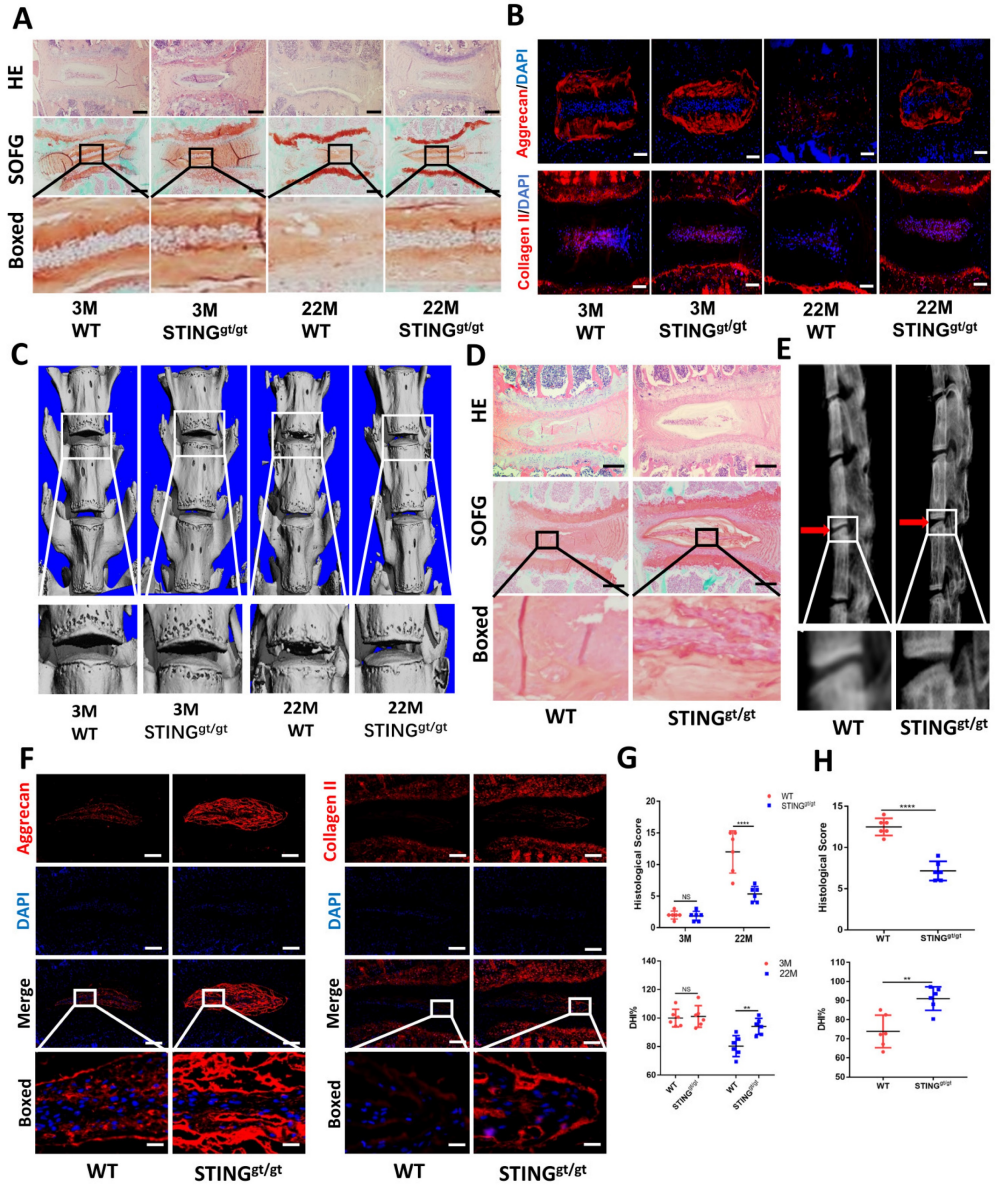
** STING deficiency protects mice from age-induced and instability-induced IDD. (A)** HE staining and SOFG staining of 3 and 22 months of WT and STING^gt/gt^ mice. **(B)** Immunofluorescence showed the change of aggrecan and collagen II in degenerative intervertebral discs in WT and STING^gt/gt^ mice. **(C)** Three-dimensional reconstructions reconstruction of the lumbar spine in WT and STING^gt^ mice at 3 and 22 months. **(D)** HE staining and SOFG staining showed changes in the IVD of lumbar vertebra instability in WT and STING^gt/gt^ mice.** (E)** X-rays showed the intervertebral disc height of lumbar vertebra instability models.** (F)** Immunofluorescence examined the changes of aggrecan and collagen II in intervertebral discs with spinal instability in WT and STING^gt/gt^ mice.** (G, H)** Quantitative analysis of histological score and disc height index (DHI%) in Figure 5A, 5C, 5D, and 5E. (The spinal level used was L4/5 level. Scale bar=50μm. Data are expressed as mean±SD. **p<0.01; ****p<0.0001).

To further confirm the involvement of STING in age-induced IDD, we generated an aging model using STING-deficient (STING^gt/gt^) mice. HE and SOFG staining of the IVDs showed significantly fewer NP degenerative changes in STING^gt/gt^ mice at 22 months than in WT littermates **(Figure 7A)**. Additionally, immunofluorescence showed a marked loss of collagen II and aggrecan and collapse of IVD space in WT mice at 22 months **(Figure 7B)**. Three-dimensional reconstructions of the lumbar spine showed significant osteophyte intrusion into the intervertebral space in WT mice but no obvious intrusion in STING^gt/gt^ mice at 22 months **(Figure 7C)**. A marked reduction in disc height and increasing histological score in WT mice but not in STING^gt/gt^ mice was observed **(Figure 7G)**. Similar protective effects were observed in STING^gt/gt^ mice using the resection-induced spinal instability model of IDD. HE and SOFG staining demonstrated severe IDD with the complete collapse of intervertebral space in WT mice but not in STING^gt/gt^ mice **(Figure 7D and 7H)**. X-ray micrographs and measurement of DHI confirmed the loss of IVD height following resection surgery in WT mice whereas minimal loss of disc height in STING^gt/gt^ mice **(Figure 7E and 7H)**. Preservation of aggrecan and collagen II content and IVD structure was found in STING^gt/gt^ lumbar spines **(Figure 7F)**. The apoptosis of NP cells was reduced, although STING can still accumulate to a certain extent after STING deficiency **(Figure S6A and S6B).** Overall, these results collectively suggest that STING is involved in the pathogenesis of age-dependent and injury-dependent IDD.

**Figure 8:**
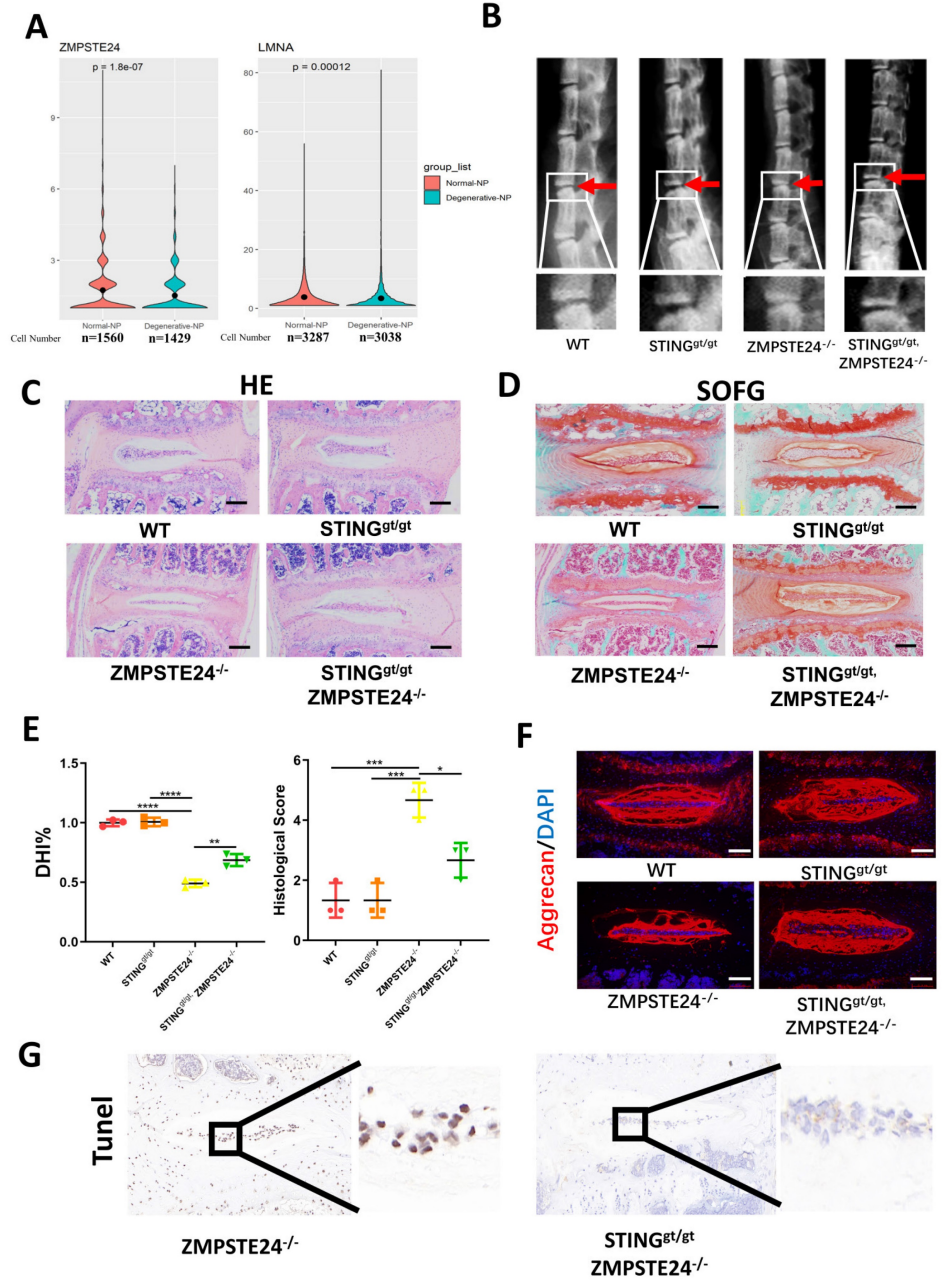
** STING deficiency protects aging-associated IDD in zmpste24 knockout mice. (A)** Single-cell sequencing showed the changes of ZMPSTE24 and Lamin A in degenerated human nucleus pulposus cells. **(B)** X-rays demonstrated that the intervertebral disc height induced by premature aging in WT, STING^gt/gt^, ZMPSTE24^-/-^ and STING^gt/gt^×ZMPSTE24^-/-^mice. **(C, D)** HE staining and SOFG staining showed changes in the IVD with the premature aging model.** (E)** Quantitative analysis of histological score and DHI% in four groups**. (F)** The changes of aggrecan in IVD in WT, STING^gt/gt^, ZMPSTE24^-/-^ and STING^gt/gt^×zmpste24^-/-^ mice were examined by immunofluorescence. **(G)** The apoptosis of NP cells was detected by TUNEL tests in IVDs of ZMPSTE24^-/-^ and STING^gt/gt^×zmpste24^-/-^ mice. (The spinal level used was L4/5 level. Scalebar=50μm. Data are expressed as mean±SD. *p<0.05; **p<0.01; ***p<0.001; ****p<0.0001).

Furthermore, zinc metallopeptidase STE24 (ZMPSTE24) is the key enzyme processing prelamin A to mature lamin A. The deficiency of ZMPSTE24 causes the accumulation of prelamin A, which leads to DNA damage resulting in dsDNA release from nuclear 25. Single-cell sequencing illustrated that ZMPSTE24 was significantly increased and lamin A was significantly decreased in the degenerated NP cells **(Figure 8A)**. Therefore, we further confirmed the role of STING in IDD through four months of WT, ZMPSTE24^-/-^, STING^gt/gt^, and STING^gt/gt^×ZMPSTE24^-/-^ mice. The ZMPSTE24^-/-^ mice showed the appearance of premature IDD. Loss of IVD height in ZMPSTE24^-/-^ mice was partially rescued by STING deficiency through X-ray measurement **(Figure 8B and 8E)**. HE, SOFG **(Figure 8C and 8D)**, and immune-fluorescence staining **(Figure 8F)** together demonstrated the deficiency of STING can alleviate the destruction of the IVD structure caused by ZMPSTE24 knockout, and protect the normal ECM components. Furthermore, the increase of dsDNA in the cytoplasm of ZMPSTE24^-/-^ mice was confirmed by immunofluorescence **(Figure S7A)**. The apoptosis of NP cells had not increased after the accumulation and up-regulation of STING in STING^gt/gt^×ZMPSTE24^-/-^ mice compared with ZMPSTE24^-/-^ mice **(Figure S7B and 8G)**. In summary, these results reiterate inhibition of STING activation and promotion of STING degradation by autophagy or gene editing method is an effective potential therapeutic intervention for the treatment of IDD. Therefore, the IVD can be protected by promoting the dysfunction of STING or its autophagy degradation.

## Discussion

This study provides evidence for activated STING and STING self-degradation induced by autophagy in the onset and progression of IDD. We showed elevation of cytosolic dsDNA, both mitochondrial and nuclear origin, in degenerative IVDs from patients with IDD as well as in IVDs from mouse models of aging, spinal instability, and ZMPSTE24 knockout-induced premature senility. Growing evidence supports cellular senescence as a major force of age-associated degenerative diseases 26. However, cellular senescence does not mean cellular inactivity, contrarily, senescent cells are highly metabolically active with increased secretion of pro-inflammatory cytokines and matrix proteases, forming a cellular phenomenon known as SASP 27. Consistent with this, we found that NP cells undergoing stress-induced senescence exhibited higher levels of metabolic and bioenergetic activity. A similar finding was demonstrated by Patil et al. in NP cells undergoing oxidative stress-induced senescence 28. Thus, we hypothesize that the adoption of SASP by NP cells is driven by the activation of up-regulated STING in response to elevated cytosolic dsDNA.

There is accumulating evidence for genomic instability in the onset of senescence, triggering an inflammatory response and driving the beginning of age-related degenerative diseases including IDD 29-31. The dsDNA is restrictively compartmentalized into specific regions in the nucleus and mitochondria. Aberrant, ectopic, or exogenous DNA in the cytoplasm are either degraded rapidly by scavenger ribonucleases or detected by cytosolic nucleic acid receptors as the danger-associated molecular pattern (DAMP) that initiates a gene expression program linked to cellular activation and cytokine production 12, 32, 33. After cytosolic dsDNA accumulation, binding of cytosolic dsDNA to cGAS catalysis the 2'3'-cGAMP which in turn engages and activates downstream key gene STING 13, 34, 35. Activated STING recruits and activates TBK1, IRF3, and P65, then causing activation of downstream inflammatory pathways 36, 37, which induces the expression of type I IFNs, interferon-stimulated genes (ISGs), and other pro-inflammatory cytokines. Previous studies demonstrated the cGAS-STING axis contributes to the pathogenesis of inflammatory and degenerative diseases (contains IDD), cellular senescence, and cancer 18-21, 35, 38. Interestingly, Ottone et al. showed that the cGAS-STING pathway only affected the structure of vertebral bone in 6-month-old mice, and had no significant effect on the senescence of NP cells 39. This result was consistent with our study, which also confirmed that STING in 6-month-old mice has little effect on disc degeneration due to the low expression level of STING and its autophagy degradation. However, in the aged mice, it was completely the opposite, the high expression and accumulation of STING significantly promoted the senescence of NP cells and eventually led to the occurrence and development of IDD. This indicates that the cGAS-STING pathway plays a completely different role in IVDs at different ages.

**Figure 9:**
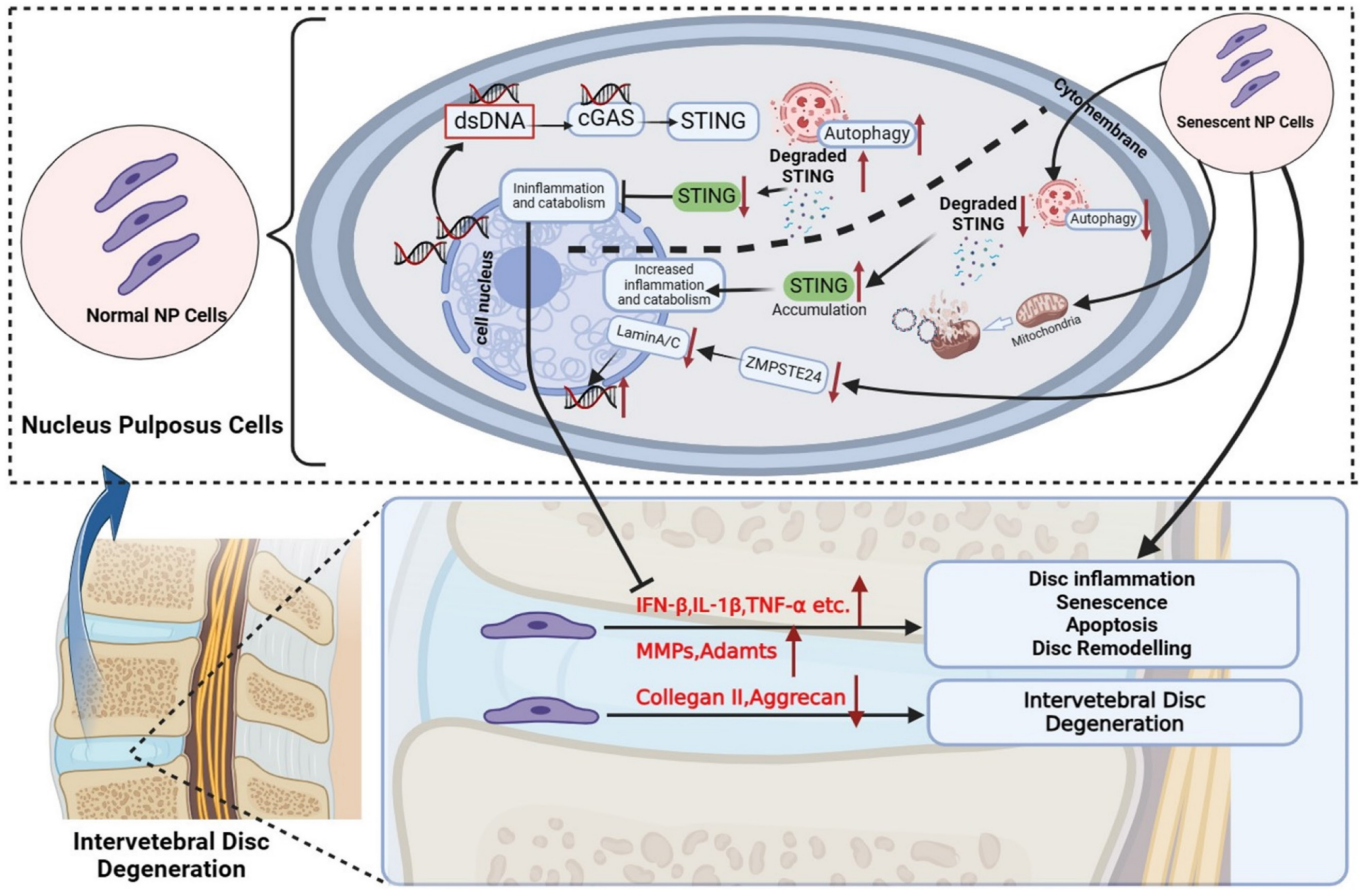
Schematic diagram of increased dsDNA (nuclear dsDNA and mitochondrial dsDNA), down-regulated degradation of STING, accumulation STING, and down-regulated ZMPSTE24 from senescent nucleus pulposus cells together created a vicious circle that significantly promoted inflammation, apoptosis, and senescence, and finally accelerate IDD. The above IDD approaches are inhibited by the autophagy degradation of STING in normal nucleus pulposus cells. (The Schematic diagram was drawn by Biorender).

In addition to mediating local inflammation and tissue destruction, type IFNs and other cytokines, namely IL-6 and IL-8, which are also produced by cells in a cGAS-STING-dependent manner can feedback in an autocrine function to induce and reinforce cellular senescence signaling. In particular, type I IFNs exhibit potent pro-senescence and anti-proliferative effects via inducing DNA damage and elevating p53 levels and signaling. In line with these effects, the activation of overexpressed STING inhibited NP cell proliferation and markedly induced NP cell senescence. This effect was correlated with elevated gene and protein expression of p53, p21, and p16 which are known for their anti-proliferative effects, promoting cellular senescence and inducing cells to a state of SASP 40. More importantly, we found that significant apoptosis could happen only when the activation of up-regulated STING in senescent NP cells. However, the increased apoptosis was slighter in normal and STING knockout NP cells. This proves the evidence that only under the co-existence of both STING accumulation and increased cytoplasmic dsDNA circumstances, NP cells have significant apoptosis. Thus, the SASP by NP cells undergoing senescence due to injury, aging, infection, etc. as well as the dysfunction of STING degradation mechanism which causes mtDNA and nDNA increase in cytoplasm coupled with the activation overexpression STING in IDD is likely a deterioration feedback mechanism that shapes the inflammatory microenvironment within the IVD that leads to onset proliferation arrest, senescence and apoptosis in senescent NP cells which progresses to IDD (**Figure 9**). The senescence of NP cells in turn aggravates the increase of dsDNA in the cytoplasm and the upregulation of STING accumulation, ultimately leading to the apoptosis of NP cells.

Autophagy is considered a highly regulated catabolic and evolutionally conserved process that not only degrades abnormal proteins but also removes dysfunctional organelles containing mitochondria, ribosomes, etc. Moreover, the immune and inflammation are also closely related to autophagy. The previous studies indicated that STING mediates autophagy to prevent pathogen infection and limit tumor growth 41. However, constant activation of the immune response is harmful to the cells, tissue, and body. Recently research revealed the degradation of STING through the autophagy-lysosome pathway to decrease the activation of cGAS-STING signaling in immune cells 22, 23. Therefore, our research further explored the relationship between the STING autophagy degradation in normal or senescent NP cells and IDD.

*In vitro*, our findings manifested that the property of STING-degradation after activation could prevent further inflammatory responses, senescence, and death in normal NP cells through autophagy. Furthermore, the phenomenon of reduced autophagy in STING was also confirmed *in vivo*. However, this phenomenon decreased in senescent NP cells causing the accumulation of STING in the degenerated nucleus pulposus. The activation and accumulation of STING were found in senescent NP cells, thus leading to more catabolism, apoptosis, inflammation, and senescence which formed a vicious circle. *In vivo* models of aging and spinal instability, the IVDs of WT littermates that exhibit age-dependent elevation in STING and dsDNA show classical signs of IDD including loss of IVD structure and ECM composition, reduced disc height and osteophyte infiltration, and eventual collapse of the IVD space. On the one hand, the cGAS^-/-^ mice model of spinal instability indicates that the loss of dsDNA receptors has a protective effect on IDD. Mice deficient in STING (STING^gt/gt^) were markedly protected from age-dependent and spinal instability-induced IDD. Defects of ZMPSTE24 lead to the accumulation of farnesylated prelamin Destroying the nuclear lamina structure and causing DNA damage and leakage easier 42. Therefore, mice knocked out by ZMPSTE24 can greatly simulate the process of cGAS activation by dsDNA, and can more directly reflect the phenomenon of reduced inflammatory waterfall caused by STING function loss. Subsequently, our data demonstrated that IDD caused by ZMPSTE24^-/-^ can also be alleviated by STING^gt/gt^ mice. Thus, our results provide further confirmatory evidence for the involvement of local inflammation caused by increased cytosolic-dsDNA and the loss of STING degradation results in up-regulated and accumulated STING on the development and progression of IDD.

This study first demonstrated that excessive dsDNA with up-regulated STING in the cytoplasm formed a vicious cycle, breaking the original STING degradation for the self-protection of NP cells. It leads to excessive accumulation of inflammatory factors in NP cells, which eventually leads to NP cell apoptosis and accelerates IDD. In normal NP cells (with non-increased STING expression), we found that indeed 2'3'-cGAMP also causes some degree of apoptosis. However, compared with cells with increased STING, apoptosis is still less. Therefore, we demonstrated that the self-degradation function of STING can still play a better role in normal NP cells, even if it is 2'3'-cGAMP with high affinity. Our above findings constituted an adequate complement to previous studies which did not mention the relationship between the changes in the protective degradation of STING and IDD. After STING was knocked down, the senescent and pro-apoptotic effects of 2'3'-cGAMP were significantly inhibited, which further proves the vital role of STING as a core protein through reduced natural degradation mechanisms and intervention manner in IDD.

## Conclusion

In summary, our current study provides evidence and first reported that the degradation of STING through autophagy and the inhibition of STING activity in normal NP cells can control aberrantly increased inflammation-induced IVD destruction. In senescent NP cells, the senescence, apoptosis, and inflammation form a vicious cycle via the activation and up-regulation of STING. Therefore, it is crucial to further explore the degradation and accumulation mechanism of STING in NP cells and clarify the regulation of STING degradation in senescent NP cells to rescue IDD.

## Supplementary Material

Supplementary figures and table.
