# Supplementary material for: Dysfunction of STING Autophagy Degradation in Senescent Nucleus Pulposus Cells Accelerates Intervertebral Disc Degeneration
Source: Int J Biol Sci. 2024 Apr 8;20(7):2370–87. doi: 10.7150/ijbs.88534 (PMC11077376; doi:10.7150/ijbs.88534)
Supplement: Supplementary file 1 — Supplementary figures and table. [file ijbsv20p2370s1.pdf]

Dysfunction of STING Autophagy Degradation in Senescent Nucleus Pulposus Cell Accelerates Intervertebral Disc Degeneration

Zhiqian Chen[1a], Chen Chen[1a], Xiao Yang[1], Yifan Zhou[1], Xiankun Cao[1], Chen Han[1], Tangjun Zhou[1*], Jie Zhao[1*], An Qin[1*]

Author affiliations

1. Shanghai Key Laboratory of Orthopedic Implants, Department of Orthopedics, Ninth People's Hospital, Shanghai Jiaotong University School of Medicine, 639 Zhizaoju Road, Shanghai, 200011, P. R. China

[*]Correspondence authors

E-mail: dr_qinan@163.com, profzhaojie@126.com, zhoutangjun@outlook.com.

[a] These authors contributed equally to this work.

Supplementary Figures and Tables

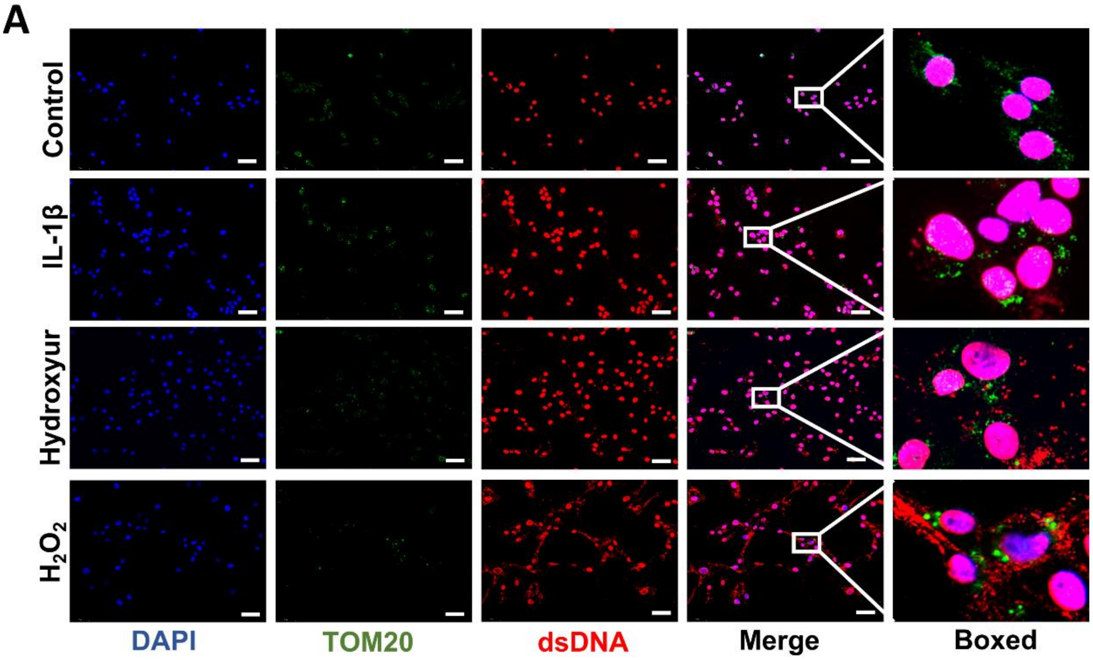

Supplementary Figure 1 Increasing cytoplasmic dsDNA in degenerative NP cells. (A) Immunofluorescence showed the distribution of TOM20 and dsDNA in the cytoplasm after being treated with IL-1β, hydrogen peroxide, and hydroxyurea. (Scale bars=50μm.)

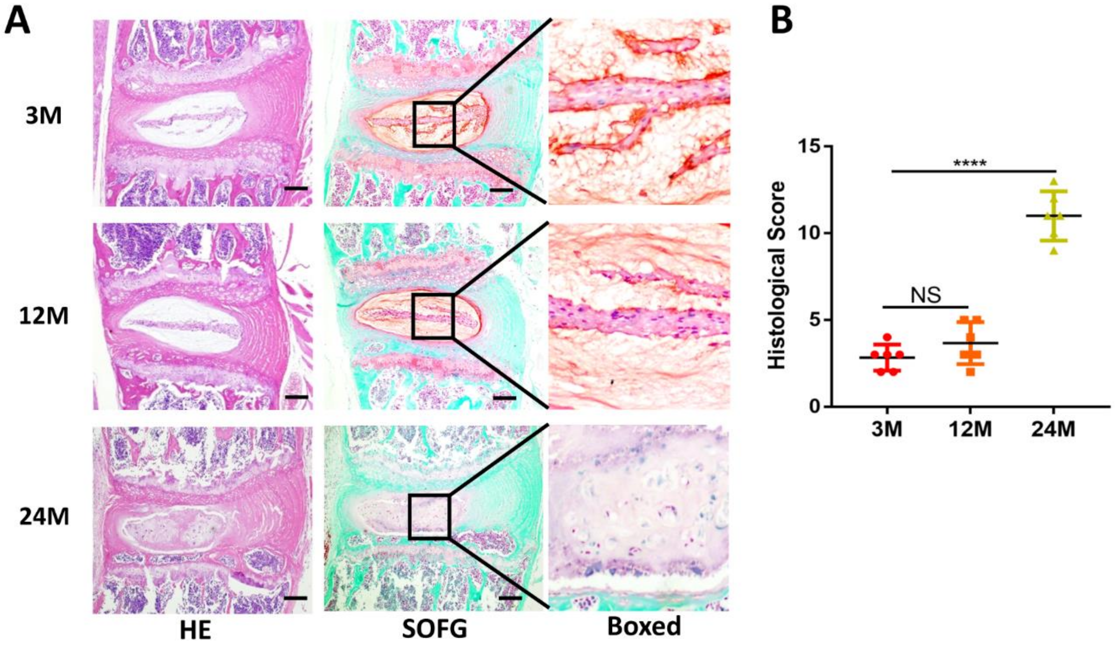

Supplementary Figure 2 Degenerative changes of the intervertebral disc with age. (A) Hematoxylin-eosin (HE) staining and Safranin O-Fast Green (SOFG) staining of 3,12 and 24 months wild-type (WT) mice. (Scale bars=50 μm.) **(B)** Histological score of intervertebral discs in mice at 3,12 and 24 months of WT mice. (Scale bar=50μm. Data are expressed as mean±SD. ****$p<0.0001$).

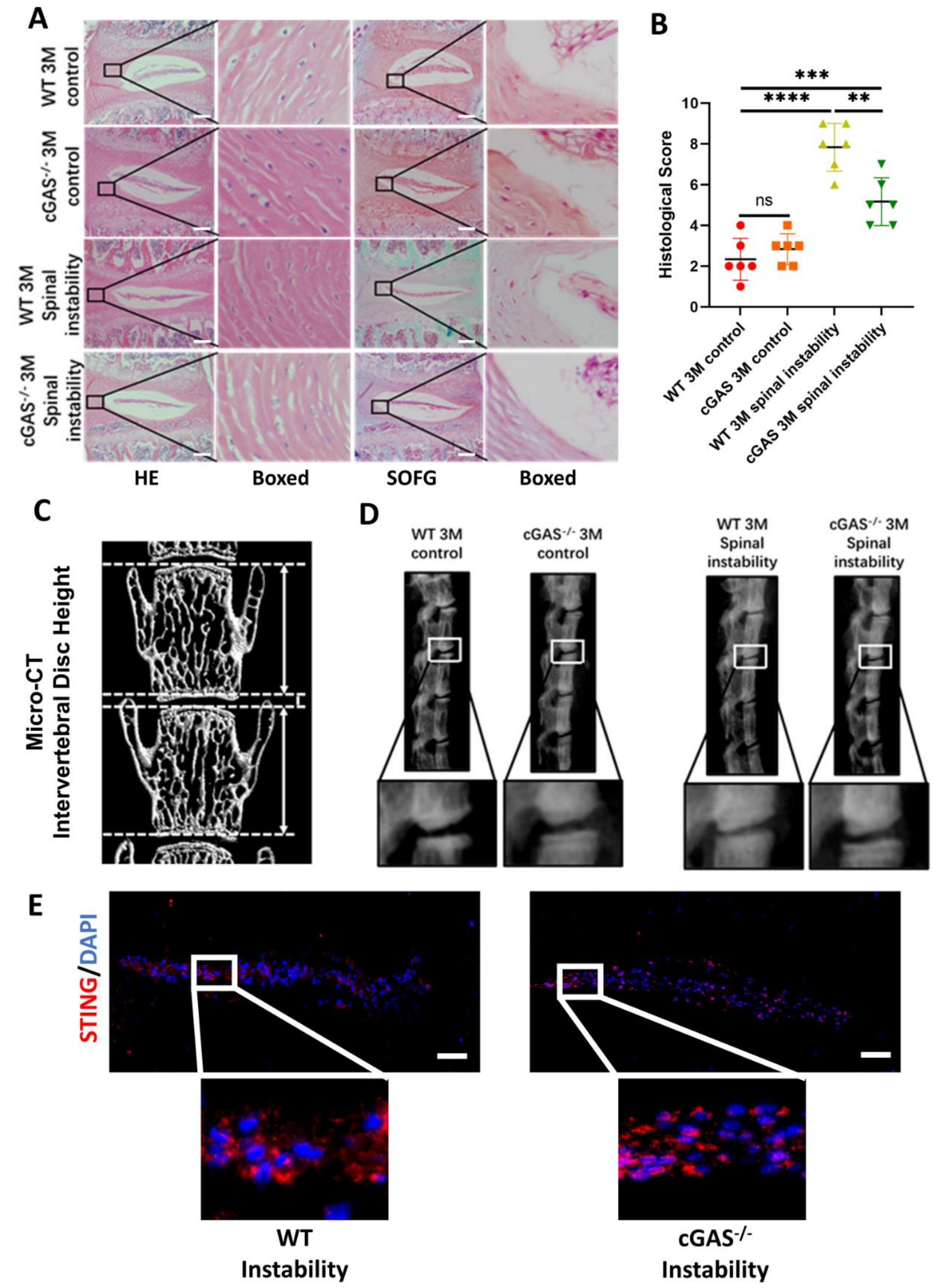

Supplementary Figure 3 cGAS knock-out protected mice from instability-induced ID and maintained the height of the intervertebral disc.

(A) HE and SOFG staining showed changes in the IVD of the lumbar vertebra instability model in WT and cGAS$^{-/-}$ mice. (Scale bar=50μm.) **(B)** Quantitative analysis of the histological score in two groups of vertebral instability models. **(C)** The calculation method of intervertebral disc height (IDH) and DHI%. **(D)** X-ray showed

the intervertebral disc height (IDH) of lumbar vertebra instability models. **(E)** Immunofluorescence demonstrated that STING expression increased in the IVDs of WT and cGAS$^{-/-}$ mice with instability model. (Scale bar=20μm.) (Scale bar=20μm.) (Data are expressed as mean±SD. $^{**}p<0.01$; $^{***}p< 0.001$; $^{****}p<0.0001$).

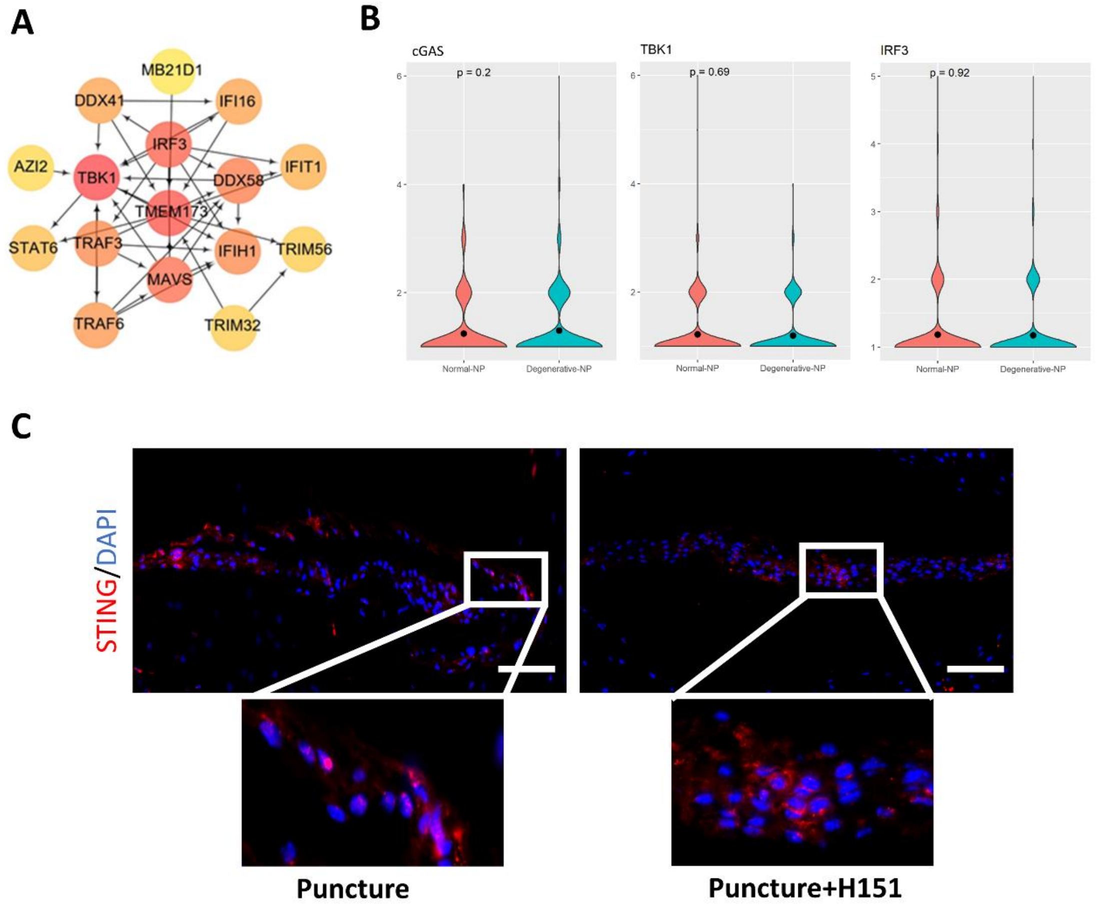

Supplementary Figure 4 Elevated expression of STING in the degenerative intervertebral disc and rat puncture model.

(A) The protein with the highest correlation with STING was enriched by the Cytoscape software. **(B)** Single-cell sequencing showed the changes of cGAS, TBK1, and IRF3 in normal and degenerative human NP cells. **(C)** The STING expression increased in the IVDs of the rat puncture model with or without injection H151 by using immunofluorescence. (Scale bar=20μm.)

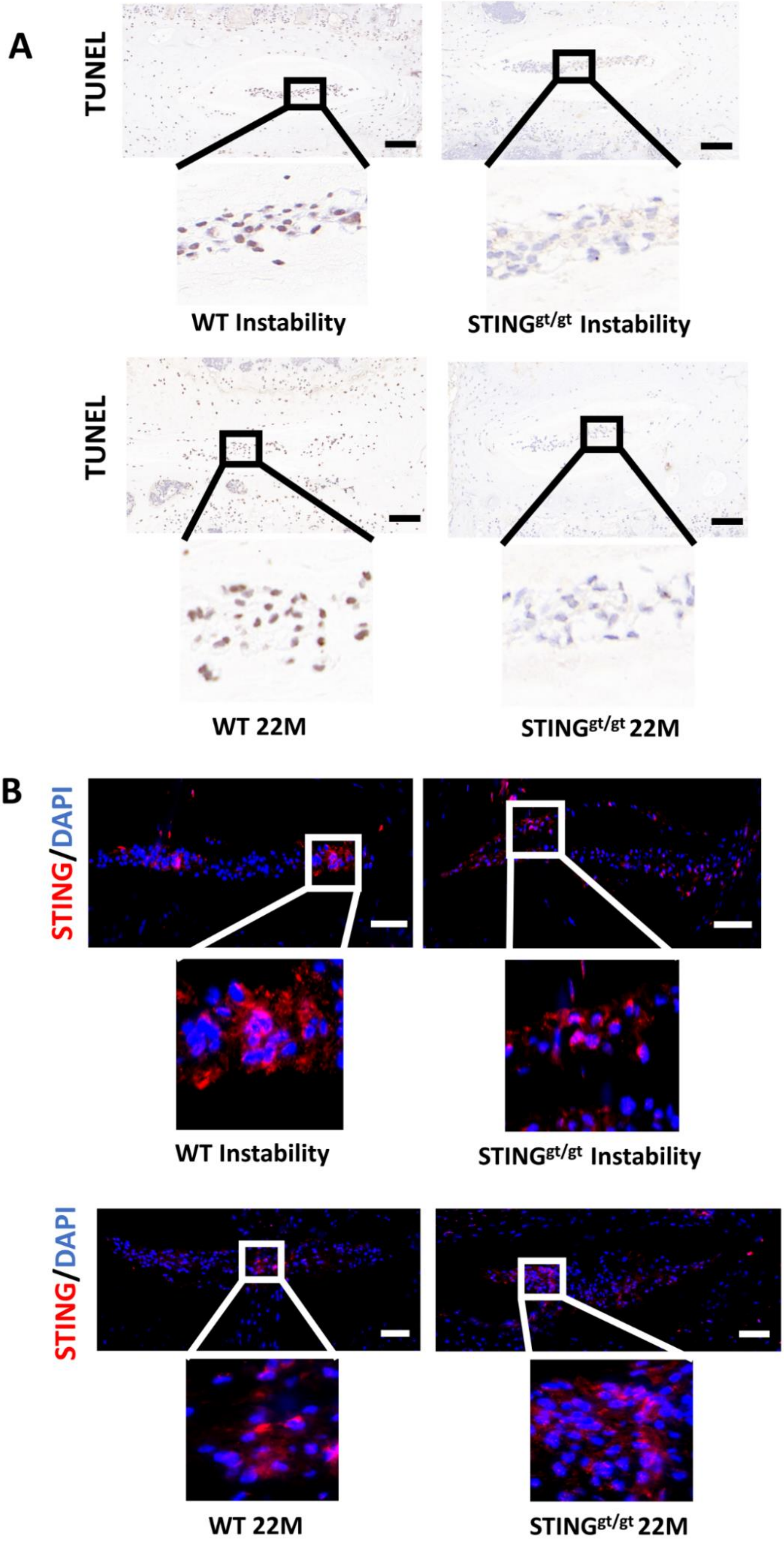
A
TUNEL
WT Instability
STINGgt/gt Instability
TUNEL
WT 22M
STINGgt/gt 22M
B
STING/DAPI
WT Instability
STINGgt/gt Instability
STING/DAPI
WT 22M
STINGgt/gt 22M

Supplementary Figure 5 STING deficiency protects mice from age-induced and instability-induced apoptosis of NP cells in IVDs.

(A) The apoptosis of NP cells in WT and STING$^{gt/gt}$ mice of age-induced and instability-induced models was detected by TUNEL tests. (Scale bar=20μm.) **(B)** The STING expression changes were detected by immunofluorescence in age-induced and instability-induced models in WT and STING$^{gt/gt}$ mice. (Scale bar=20μm.)

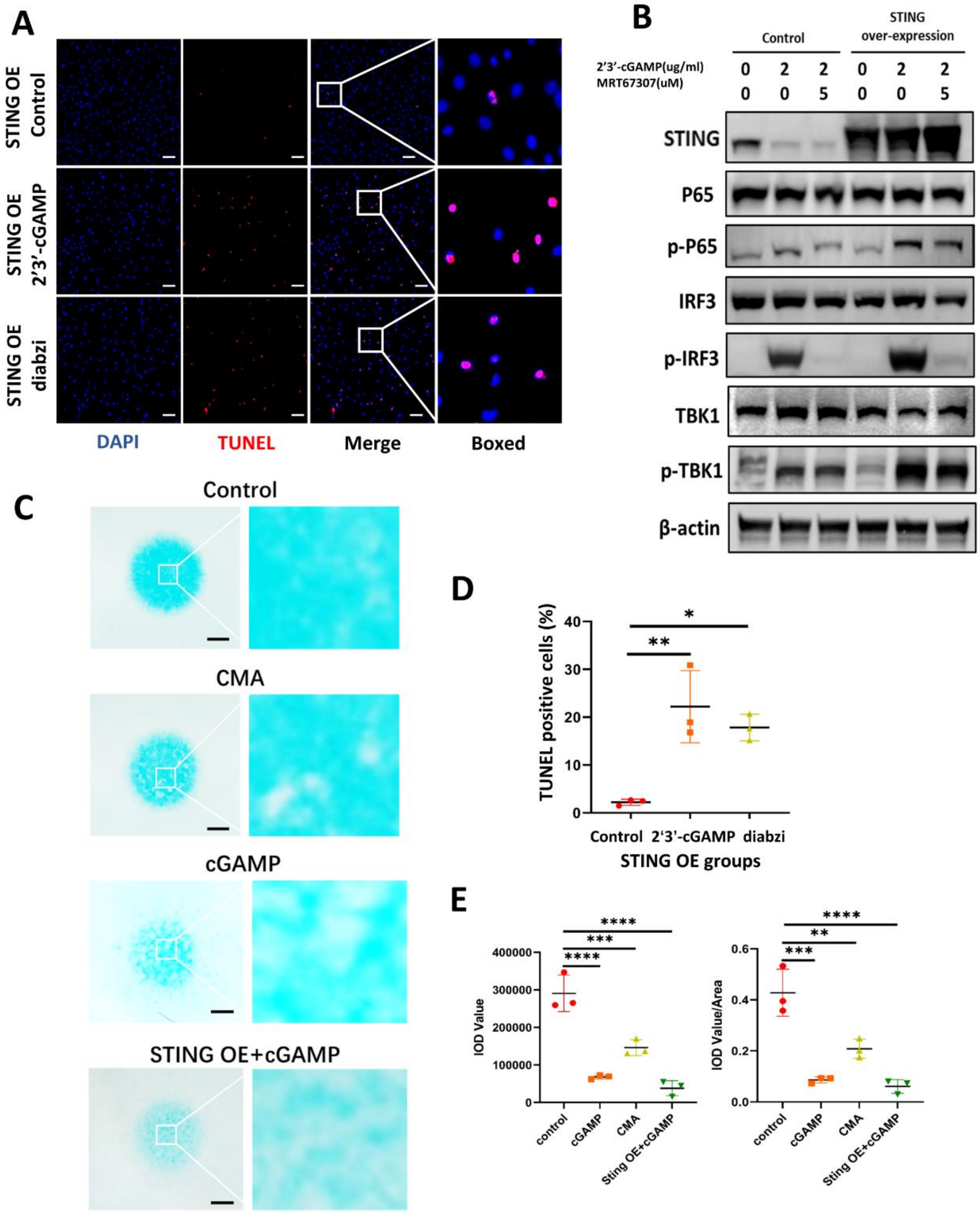

Supplementary Figure 6 Up-regulated STING in the degenerative intervertebral disc promotes apoptosis, activation of inflammatory pathways, and increases catabolism.

(A)TUNEL staining showed that the number of apoptotic NP cells increased after up-regulated STING activation. (Scale bar=50μm.) **(B)** Western blot examined the changes

of phosphorylation of P65, TBK1, IRF3 in NP control cells and STING overexpression NP cells after stimulated with 2’3’-cGAMP, 2’3’-cGAMP+MRT67307 for 2 hrs. **(C)** Acian blue staining showed the changes in the extracellular matrix (ECM) of NP cells high-density culture after treated with cGAMP, CMA and over-expressing STING+cGAMP (Scale bar=5mm.) **(D)** Quantitative analysis of the TUNEL-positive NP cells stimulated with 2’3’-cGAMP and diabzi in Figure S5A. **(E)** Quantitative analysis of the IOD and IOD/area of Alcian blue staining in supplementary figure S5C. (The cells used were the rat nucleus pulposus cell line. Data are expressed as mean±SD, *P<0.05; **p<0.01; ***p< 0.001; ****p<0.0001 compared with controls).

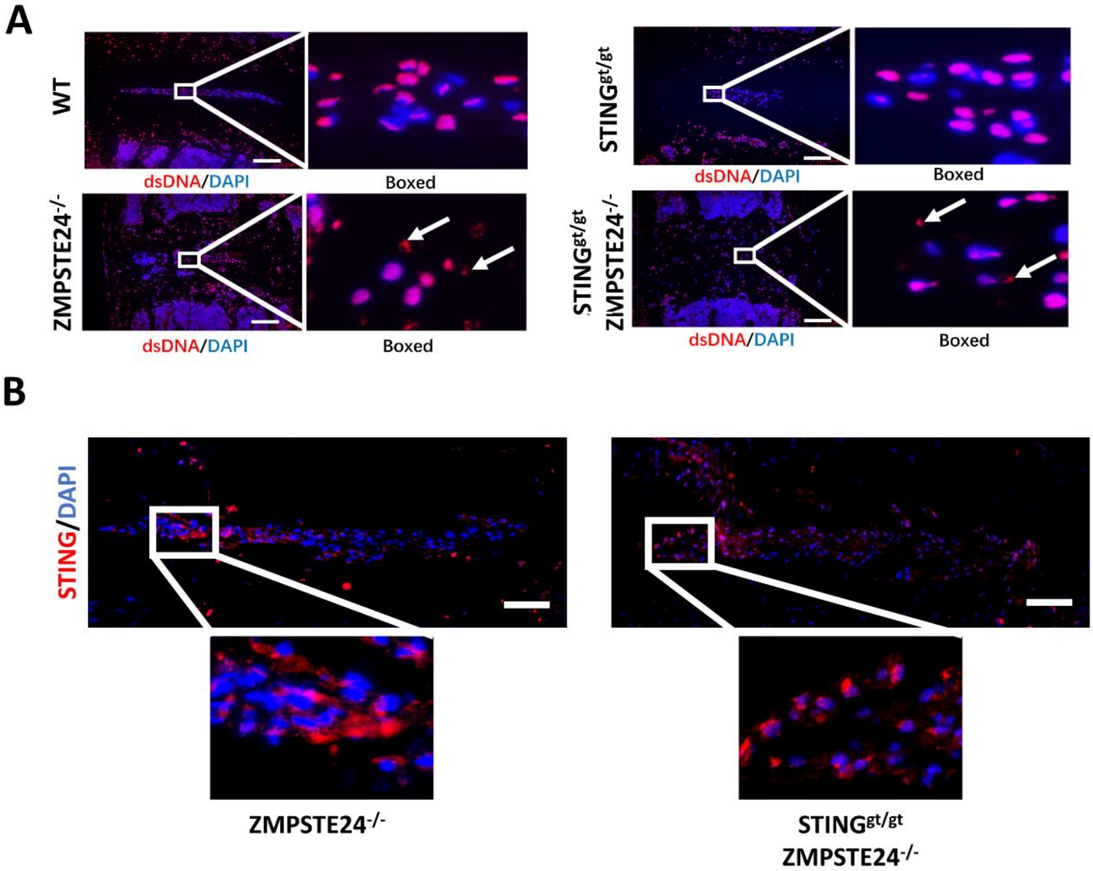

Supplementary Figure 7 Cytoplasmic dsDNA was significantly increased in $ZMPSTE24^{-/-}$ mice.

(A) Immunofluorescence demonstrated the distribution of dsDNA in IVDs in WT, $STING^{gt/gt}$, $ZMPSTE24^{-/-}$ and $STING^{gt/gt}$×$zmpste24^{-/-}$mice. (Scale bar=50μm.) (B) The STING expression changes were detected by immunofluorescence in $ZMPSTE24^{-/-}$ and $STING^{gt/gt}$×$zmpste24^{-/-}$mice. (Scale bar=20μm.)

Supplementary Table Sequences of Primers for Quantitative Real-time PCR.

Primer sequence			
**Rat**	ACTB	F	AGTGTGACGTTGACATCCGT
**Rat**	ACTB	R	CTATGGGTCCAGGCTAAGGC
**Rat**	B2M	F	AAAAGGCCGATCCGTAGTGC
**Rat**	B2M	R	TCCGGCACTTAGTGTGCATC
**Rat**	GUSB	F	AAGCCAATTATCCAGAGCGAGT
**Rat**	GUSB	R	GGCCACAGTGTGTAGGCTTAG
**Rat**	mtND1	F	ATAAGCGGCTCCTTCTCCCT
**Rat**	mtND1	R	GAATGGTCCTGCGGCGTATT
**Rat**	mtCYTB	F	AGCAACCCTAACACGCTTCT
**Rat**	mtCYTB	R	ATGGGATTTTGTCTGCGTCG
**Rat**	mtCOX1	F	AGCAGGGATACCTCGTCGTT
**Rat**	mtCOX1	R	CAAGGACGGCCGTAAGTGAG
